# Supplementary figures and images for: Short-term neuronal and synaptic plasticity act in synergy for deviance detection in spiking networks
Source: PLoS Comput Biol. 2023 Oct 13;19(10):e1011554. doi: 10.1371/journal.pcbi.1011554 (PMC10599548; doi:10.1371/journal.pcbi.1011554)

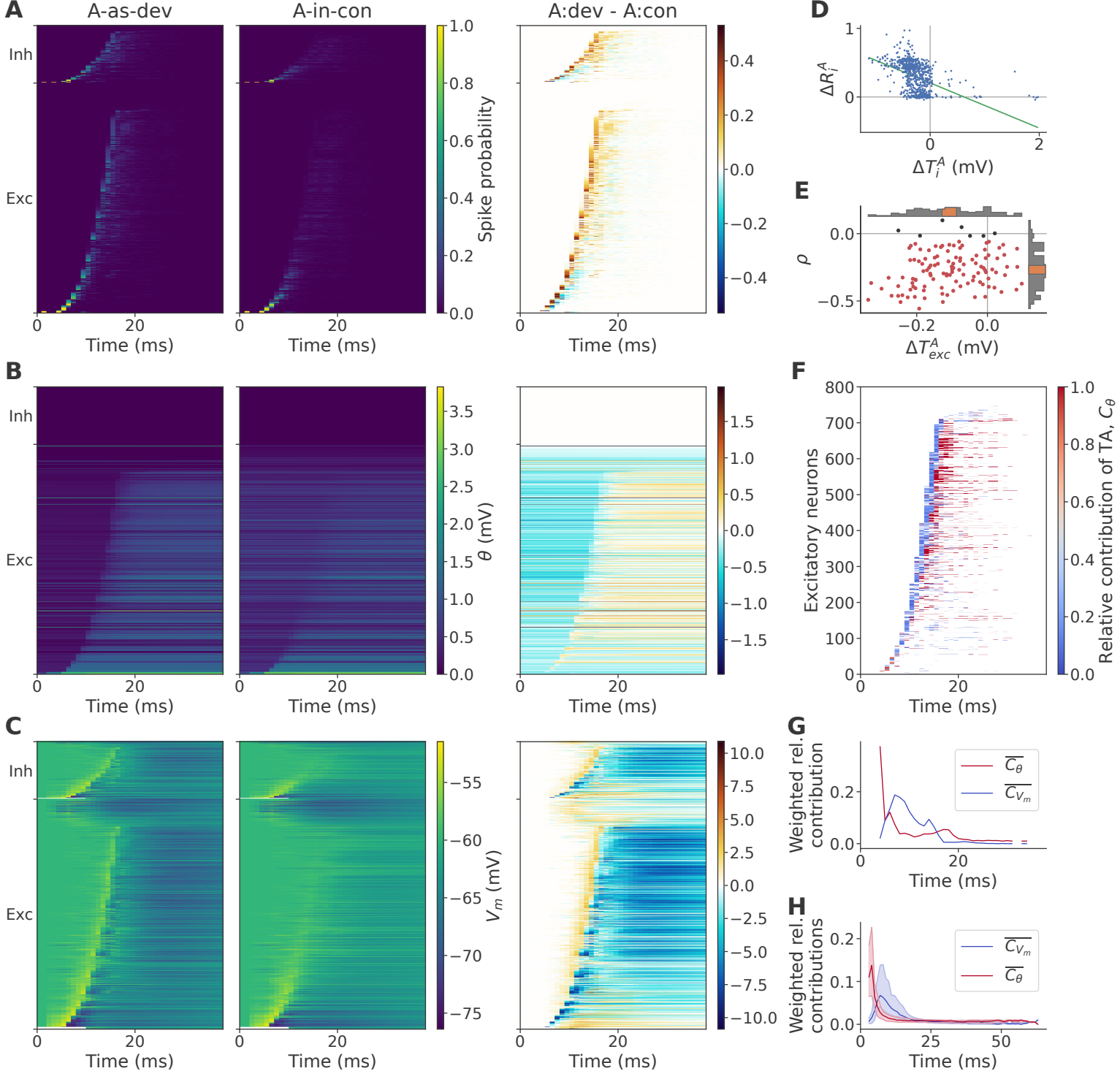

Supplement: S1 Fig — (PDF) [file pcbi.1011554.s001.pdf]

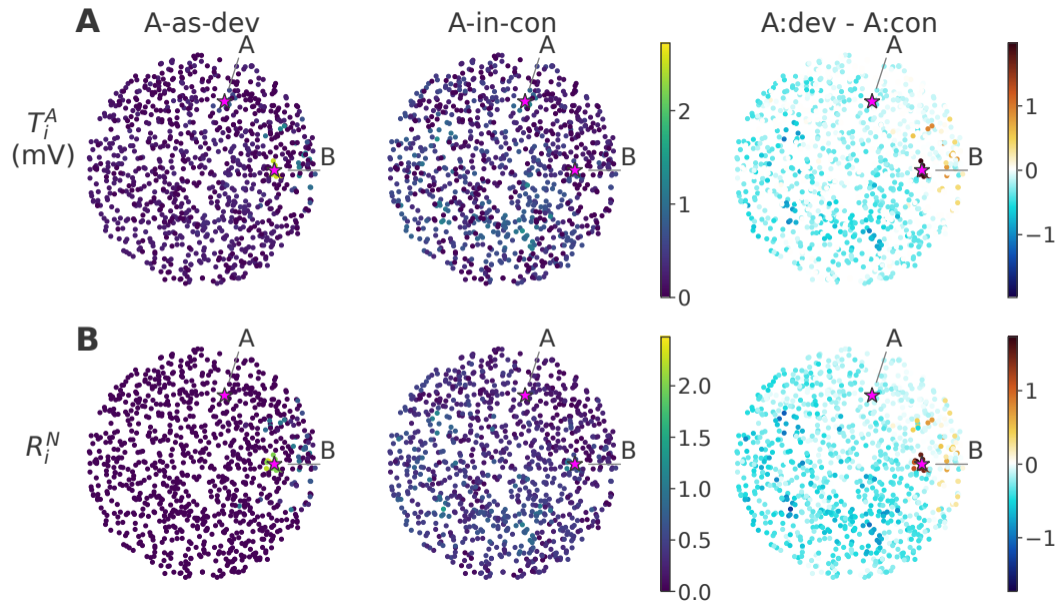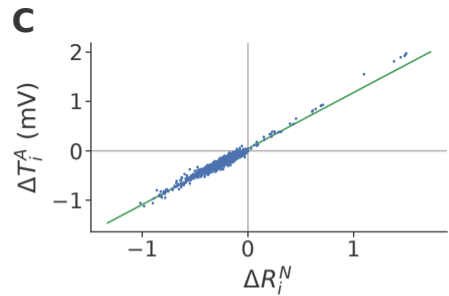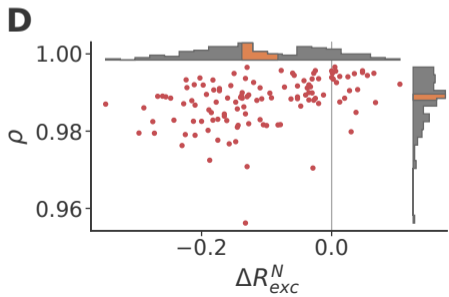

Supplement: S2 Fig — (PDF) [file pcbi.1011554.s002.pdf]

**A**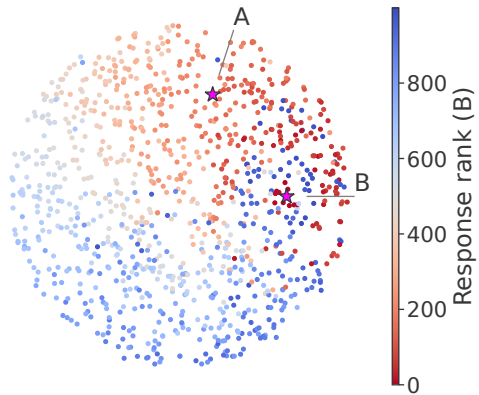**B**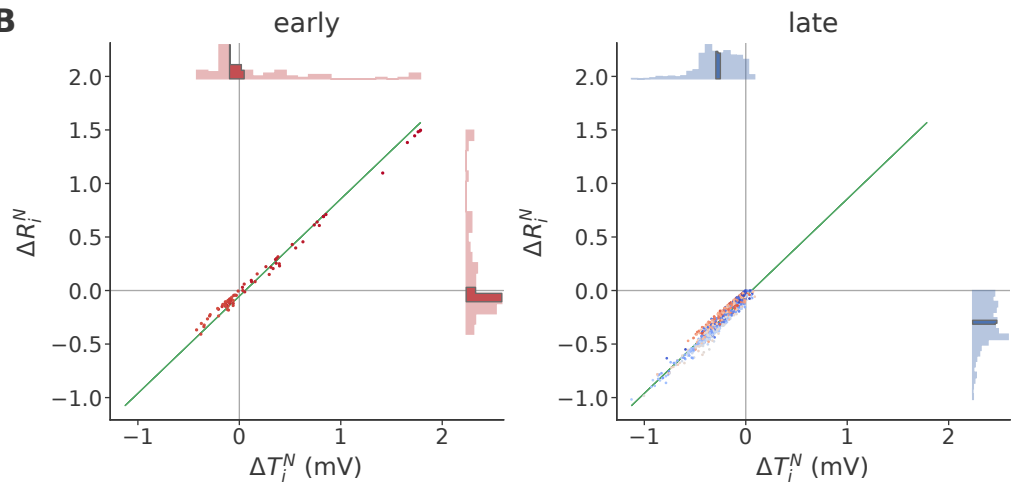**C**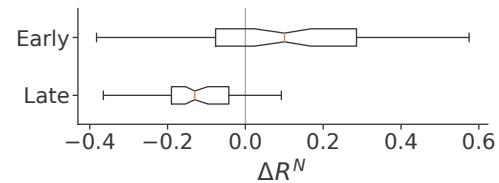**D**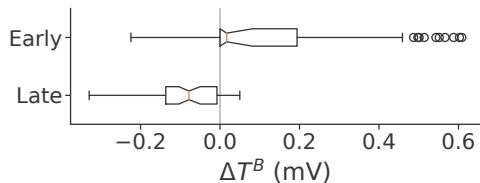**E**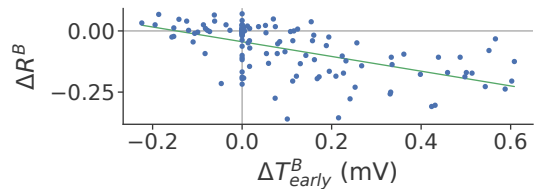

Supplement: S3 Fig — (PDF) [file pcbi.1011554.s003.pdf]

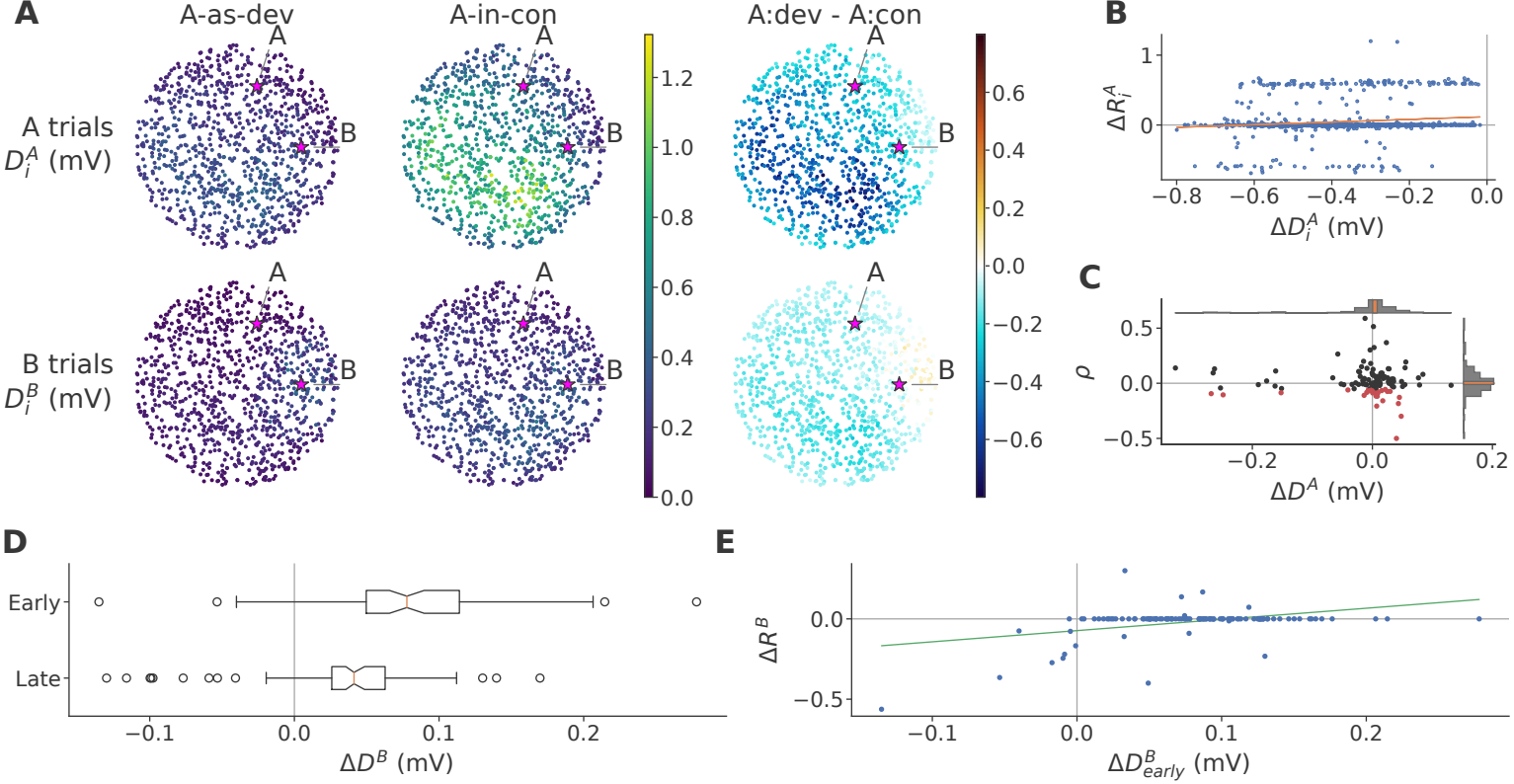

Supplement: S4 Fig — (PDF) [file pcbi.1011554.s004.pdf]

A-as-dev

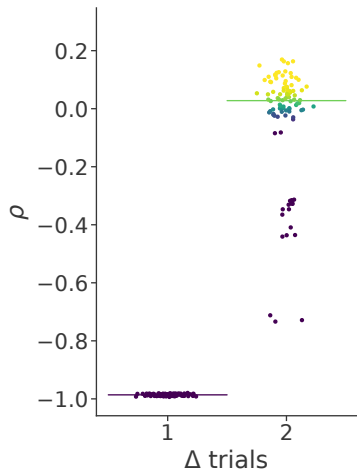

A-in-con

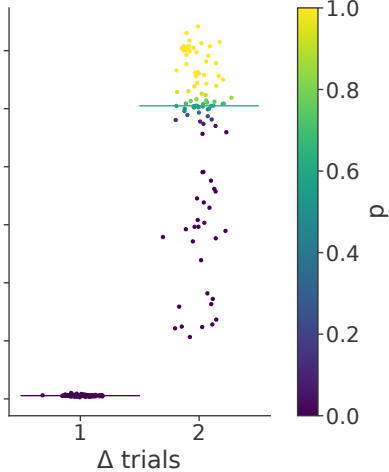

Supplement: S5 Fig — Each marker represents the coefficient ρ of the Pearson correlation between the spike counts in a target trial and the corresponding values xi at the beginning of the immediately following trial (Δ trials = 1) or after one intervening trial (Δ trials = 2). Recall that x is reduced upon spiking, which implies that an effective correlation is negative. Color represents the p-value of the alternative hypothesis that ρ < 0. Horizontal lines indicate the median ρ and p. Horizontal jitter is introduced to aid visual separability of the markers. (PDF) [file pcbi.1011554.s005.pdf]

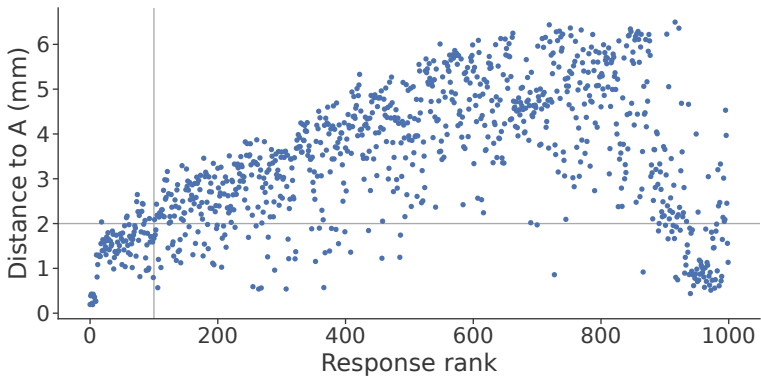

Supplement: S6 Fig — Distance to stimulus site A plotted against the response rank (see Fig 6) in the sample network. Vertical and horizontal grey lines represent the cutoffs for the “early” subset (Fig 6) and the “local” subset (Fig 9), respectively. Notice that, while many neurons are both early and local, or both late and global, a substantial number of neurons are early and global, or late and local. (PDF) [file pcbi.1011554.s006.pdf]

A:std

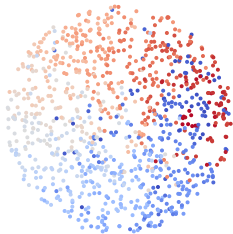

A:con

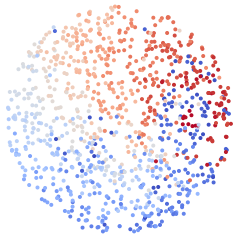

A:dev

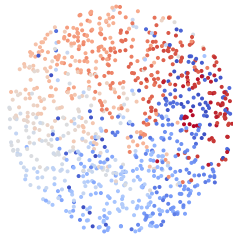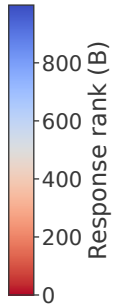

Supplement: S7 Fig — See Fig 6A. (PDF) [file pcbi.1011554.s007.pdf]

**A**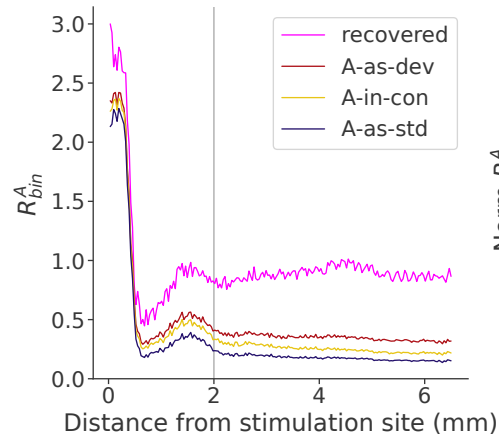**B**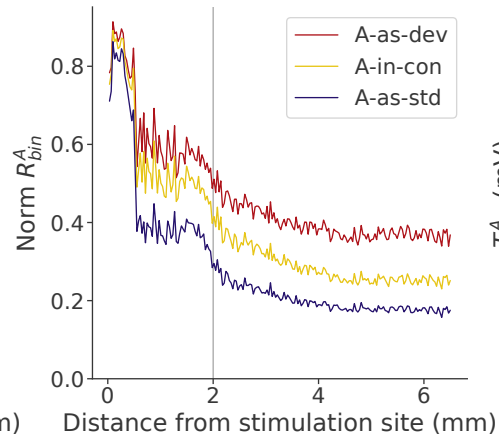**C**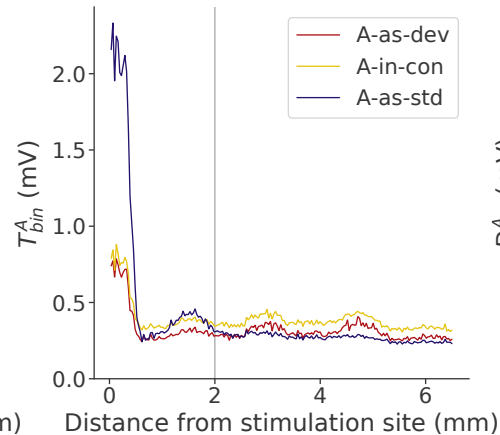**D**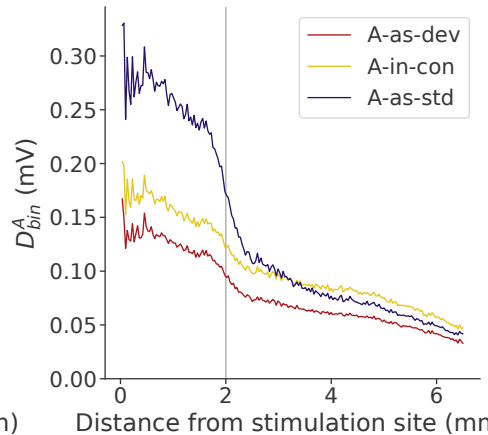

Supplement: S8 Fig — A: Unnormalized response size R, mean across all datasets. The three sequence averages, which show a pronounced local maximum around 1 mm to 2 mm, are shown in addition to the recovered response (i.e., the response to stimulation in A outside of sequence context), which was used for normalization (B, Fig 9A). B-D: As in Fig 9A–9C. (PDF) [file pcbi.1011554.s008.pdf]
