# Supplementary material for: Short-term neuronal and synaptic plasticity act in synergy for deviance detection in spiking networks
Source: PLoS Comput Biol. 2023 Oct 13;19(10):e1011554. doi: 10.1371/journal.pcbi.1011554 (PMC10599548; doi:10.1371/journal.pcbi.1011554)
Supplement: Attachment — Submitted filename: Reviewing of Kern and Chao Manuscript for PLoS Computational Biology.docx [file pcbi.1011554.s009.docx]

**Review of Kern and Chao Manuscript for PLoS Computational Biology:**

**“Short-term neuronal and synaptic plasticity act in synergy for deviance detection in spiking networks”**

This modeling work By Kern and Chao presents an investigation of possible mechanisms for the neuronal process of deviance detection, which is an important building block of sensory processing and scene analysis, and has been studied extensively in the context of human cognition, as well as in animal models.

This work introduces into the field of modeling deviance detection and related phenomena the well-known neuronal property of threshold adaptation. The authors argue correctly that this inherent neuronal process has so far been left out of theoretical studies, even though it is obviously present in the systems being investigated, and as a dynamic adaptation process is likely to have some involvement in the way neuronal systems adapt to frequent and regular stimulation.

The progress presented in this work is not conceptually innovative, as it takes the logical next step that is needed in the development of its field. However, there is merit in the authors’ choice of model and in their construction of the neural network that they used. They elegantly apply a network of integrate-and-fire neurons, which allows an explicit inclusion of both threshold adaptation and synaptic depression as well as access to the dynamics of single spikes, while avoiding details that are probably unnecessary for investigation at the level of network responses. Furthermore, they employ a simple connectivity scheme and avoid an explicit description of any specific stimulus space, sensory system, or brain area, demonstrating the phenomenon on the general, conceptual level and extracting principles that are likely generalizable to different occurrences of deviant detection in the brain.

The investigation presented in this manuscript is well conducted, delving into the complexity of having two mechanisms with similar dynamics and outcomes performing side-by-side and in synergy, and providing rigorous evidence for its claims. It produces a solid demonstration that threshold adaptation should be included in models set on investigating deviance detection, and of its workings and impact.

My recommendation is that this manuscript be accepted pending minor revisions. The general points of which I am critical are that this work does too little to support its choices of network structure and parameters on physiological evidence from real neuronal networks operating in the brain that show deviance detection, and that it does not do enough to relate its findings to the actual phenomenology found in experiments (e.g. in the strength of deviance detection and the temporal profile of responses to truly deviant vs. infrequent stimuli). In addition, there is insufficient investigation of the impact of choices that were made in constructing the model, or justification for not investigating it. As a result, the authors’ claim that threshold adaptation is a major driver of deviance detection, with synaptic depression acting to support and enhance it, is put in doubt, as it is not clear how much this claim is specific to the way the network was constructed or to the stimulation paradigm that it was presented with. An acceptable solution to this would include either of the following: (a) simulations where the network is stimulated at different frequencies, with an investigation of the resulting dynamics to demonstrate whether the dominance of threshold adaptation applies also for higher-frequency stimulation; or (b) grounding the choices of network parameters and connectivity in evidence from the experimental literature, to show that the brain area(s) being studied, when modeled with reasonably close parameters and stimulated similarly to experimental studies, are plausibly relying on threshold adaptation more than on synaptic depression for their deviance detection. I have listed in my comments additional doubts and questions that I had about the operation of the model with different choices of parameters, e.g. of connectivity and of inhibition. While I would appreciate consideration of these and possibly simulations to address them, I would not require full investigation of those points and characterization of their results in the present context, as they would increase the scope of the work by too much, and are possibly a matter for potential independent follow-up studies where they can be granted the focus and the thoroughness they deserve.

**Comments on the Manuscript:**

**Major Comments**

1. In the author summary, and then in lines 50-55, the authors mention that improved understanding of deviance detection could help develop treatments for several disorders. Would they be able to elaborate, or hypothesize, on how the understanding gained in the present work sheds light on the aberrant processing or the symptoms in the said disorders, and might guide the development of new treatments? (this does not need to go into the paper, unless the authors would like to mention it somewhere).
2. In line 59, true deviance detection is described as an “enhancement” of a response in one condition compareג to another. However, the responses to true deviants are not enhanced by the regular background, but merely less reduced than responses to the same stimuli when they appear over an irregular background.

In general, throughout the manuscript the authors use descriptions like “increase”, “reduced” “reduction”, etc. in comparisons of responses and quantities that are taken from the different stimulation sequences. This can imply that there is a dynamical process that leads from one situation to the other, which is confusing. It would be best to replace this terminology with descriptions like “higher/lower than X”, “strong(er)/weak(er) than X”, etc., making sure to name both of the quantities being compared (for negative quantities, “more negative/less negative” to avoid confusion).

Examples include “increase” in line 241; “decrease” in line 277 (it’s not that T is decreased, just that it is lower/less increased in the dev sequence than in msc); “reduction” in lines 294, 316; “increasing” in lines 343, 362 and “decreased” in lines 344, 353; “increased” in lines 390, 391; multiple instances in lines 393-418; “reduction” in lines 436, 456, etc. (also line 657).

3. Line 75 (and also 583-584): It is not accurate that TA was rejected a-priori as a mechanism for DD. Rather, TA was in a way skipped in the consideration of mechanisms for DD, since it was already rejected as a mechanism for SSA: Yarden and Nelken mention that intrinsic neuronal plasticity cannot easily explain SSA, while reviewing the reasons that models that preceded theirs have used STD and not TA. However, those previous models were all of the feed-forward type, so TA was taken out of the equation already when the field was focusing on feedforward models, and was overlooked as a potential mechanism when the architecture is of a recurrent network. The results of Yarden and Nelken demonstrated that in fact, TA should have been reconsidered: once a recurrent network is used, that has some compartmentalization, TA should support SSA and DD in much the same way that their cortical columns do by STD, since each of their columns essentially served as a unit that is mostly adapted by one stimulus (and more weakly adapted by other stimuli) – a manner of adaptation that could just as well be caused by TA. This was of course not recognized in their paper. The details of this course of development do not need to be laid out in the present manuscript, but consider refining the point about how TA was rejected or overlooked.
4. Line 99: Why were the inhibitory synapses not made to have STD? It is known that the synapses that fast-spiking (PV) neurons make on excitatory neurons in cortex are depressing. Can the authors provide the reasoning behind not using STD in inhibitory synapses (esp. since they are explicitly modeling fast-spiking neurons)? Can they give a hypothesis about how leaving this physiological detail our of their model might have affected their results or their applicability to cortical networks (or test how they might change if this feature is added)?
5. Line 102: How were tau_x and U chosen? If they were based on physiological data (they are indeed correct for auditory cortex, for example), please cite the sources for these values and what parts of the brain they might simulate (in another place, line 631, this model is stated to correspond to, or also to, subcortical areas – do these values apply to those areas? Would they need to be modified for that?).
6. Line 102+3 (there are several lines between 102 and 103): What is the reason for making all of the inhibitory neurons fast-spiking (and non-adapting)? Can the authors provide a hypothesis (or a test) about the impact of non-fast spiking neurons in this network on DD?
7. On a side note, the model presented here appears suitable to test the possible effect or role in DD of facilitating synapses, both between excitatory neurons (an interesting prediction is that they might actually enhance the responses to infrequent stimuli, following Mongillo et al., Science 2008), and from inhibitory to excitatory neurons (namely the facilitating synapses that Martinotti cells make on the excitatory neurons). The authors are encouraged to consider testing these things in their model, but of course that would substantially increase the scope of this work, and may be more appropriate for a different paper to follow up on this one.
8. Line 103: Given that tau_theta is much longer than tau_x, it appears almost inevitable that TA would prove to have a greater effect on DD than STD. However, this also depends on the parameters of the paradigm used to stimulate the network. Would the expected relative contributions of STD and TA to DD be different with shorter intervals? Have the authors tried to run the paradigm with shorter intervals between the stimuli, or can they provide an example that demonstrates similar or different relative contributions (e.g., runs of the sample network stimulated with shorter intervals, ablated as in Fig. 3)?
9. Line 114: Could the authors provide an explanation or reference for the choice of having each neuron form 50 synapses, and the ranges of the different synapses? What is the resulting probability of connection and how does it relate to known connectivity patterns in the brain? What area of the brain are these parameters supposed to model (and how would they be different for other areas that the authors claim to capture with the model or its principles, like cortical/subcortical areas)? How would the authors expect different choices to affect the DD in the network?
10. Line 114: Why are all synapses chosen to have a weight w = 1? Why not a gradual decrease in weight with distance? Why are the inhibitory and excitatory synapses of the same weight? Was this chosen empirically for maintaining a specific balance (and if so, what were the criteria?), or depends on the connectivity pattern – or based on physiological data? What would be the effect on DD of choosing to have differently-weighted excitatory and inhibitory synapses, or randomly weighted synapses with a profile of the strength decaying with distance between the somata?
11. Line 119: How was the number of stimulated neurons chosen? And the strength of this stimulation? Is stimulating 10 neurons at 2-3 spikes each meant to model or is based on data from any specific physiological system? If so, please provide references. What would be the effect, and the consequences for DD, of having the stimulus evoke responses of different magnitudes in an area out of the network according to a tuning curve? Finally, are the stimulated neurons only excitatory neurons, or also inhibitory? Please specify in the text.
12. Stimulation of this model was performed by a one-time increase in the excitatory conductance. What would be the effect on the network and on DD of a longer stimulus?
13. Lines 137-138: Was there any difference in terms of DD or otherwise between the two stimulus pairs used?
14. Fig. 2: The responses shown here are averaging over the whole network. What experimental paradigms from the literature should this be compared to?
15. Fig. 2: What do the responses of single neurons in the network look like? Do they all have dev > std and dev > msc? Can the authors provide a distribution or a scatterplot, perhaps from the sample network?
16. Fig. 2: Do the different time-profiles found here for the dev, msc, and std responses resemble any experimental results from the literature?
17. What is the significance of the finding that most networks had a positive DDI? I am presuming that the authors take this to mean that their model can generate DD without a significant dependence on the detailed connectivity. However, for establishing that TA can generate DD, wouldn’t it suffice even if only a minority of networks were able to show DD?
18. Why is STD so ineffective in generating DD in this model? Yarden and Nelken’s model had only the STD and was able to generate DD, albeit not nearly as strong as that found here (though they did not compute a DDI nor took the response over the whole network).
19. Line 220: I would like to see a comparison of the DDI in this model with similar quantities from experimental studies (could be here or in the discussion section).
20. Lines 262-278: This paragraph describes how the authors verified that lower T leads to larger R. I appreciate the thoroughness, but is this not an inherent feature of the model? Could it have possibly been otherwise?
21. Lines 279-292: The calculations described in this paragraph, and the reasoning behind them, are very unclear. I would appreciate an explanation of why the measures described there were used and how they were chosen, including why there was weighting by the magnitude of the response difference (was it not “normalizing”? Was the response difference used to multiply or divide the terms?). In line 286, it is not at all clear what the “relative terms” plotted in Fig. 4G-H are. If the quantities described in this section are better explained using formulas, those would be welcome. Please consider coining your terms for these quantities. This would make it simpler to have them explained in more detail in the methods section, and refer to them by name in the results section, with at most a general and short mention of their purpose and nature.
22. Lines 443-455: This explanation can probably be transferred to the methods section, as it is too technical for the results section.
23. Line 449: Where does the value of k = 1.4 mV come from?
24. Lines 459-469: The explanation here is hard to follow, somewhat meandering. Please rephrase this passage to make it more clear.
25. Lines 474-475: Can the authors explain the situation in the minority of networks, where greater depression in dev did not predict a lower response?
26. Line 477: Why is there no negative dependence in the model with STD only?
27. Line 483, and perhaps elsewhere: The phrasing “neurons… showed greater depression…” is somewhat confusing. Perhaps it could be stated in a different way, or given another term rather than simply “depression”, to maintain the distinction that this quantity that is being referred to is a measure for the impact of pre-synaptic depression on a post-synaptic neuron.
28. Line 502, and elsewhere: “Suppression” in the context of STD is a bit misleading, since there is no active factor suppressing the responses. Please consider alternative terms (e.g. depression, depletion, habituation, adaptation,…). This also applies to TA, in fact. Nevertheless, it is also acceptable to keep “suppression” as it is used now if the authors view it as preferrable to alternative terms.
29. Lines 505-507: Here, and elsewhere, the authors present the claim that adding STD to a model increases its DDI that is generated by TA. However, I am concerned that this way of things comes about from constructing a network, or using a stimulation paradigm, where STD does not in itself produce DD, contrary to previous modeling work. While the synergy of the two mechanisms that is demonstrated and explained in this present work and mentioned in lines 512-515 is well appreciated, I am wondering if, had the authors started out with a different network or paradigm, they would have reached the opposite conclusion, that it is TA that increases DD when added to a network with STD.
30. Fig. 9A: Is it correct to infer from the curves that there is no DD at the stimulation site? What is the significance of the network only being able to detect true deviance outside of a stimulus’s representation?
31. Fig. 9B: What gives rise to the spatial wave seen in B?
32. Fig. 9, A-C: What causes the local maxima found just on the left of the divider line in both A, B, and C?
33. Lines 529-541: Do the local and distal subsets correspond to the early- and late-responding neurons from earlier?
34. Discussion – 1^st^ section and line 554 (and also line 212): I would like to note that “learning” is a term not usually used for the processes happening in neuronal networks (as opposed to artificial neural networks), where the processes underlying learning are referred to by terms like plasticity, adaptation, encoding, etc. Learning would be the process happening on a higher or more abstract level, referring to the way an organism acquires information and is able to use it later on, or adapts to a certain environment or process in a way that changes its behavior. I do not necessarily require the replacement of this word, but please give thought to its use.
35. Lines 606-608: Were the occasional large responses to std in this model observed in different single neurons on different trials, or were they synchronized responses of the whole population?
36. Lines 611-612: What is meant by “very minimal tuning and exclusion criteria”?
37. Lines 611-612: It is not clear to me why the authors expect that their model has a certain level of robustness, and to what parameters they expect it to be robust. Please elaborate. The parameters chosen here are very specific; I can see how robustness to parameters of the stimulation paradigm (which was not tested, but is easy to test) could easily be explained by the very long tau_theta.
38. Line 613: Is this sentence stating that the present model has looser connectivity requirements than other models, and is thus applicable to a wider range of architectures, or that if the model’s connectivity requirements be made looser, it will be applicable for a wider range of architectures?
39. Lines 614-616: What structural constraints of their model do the authors consider as significant for the issue of judging what mechanism gives rise to DD? Does their model change its DD properties if the parameters of the lateral inhibition (connectivity of their inhibitory neurons) are changed?
40. Lines 617-618: What feedforward adaptation does the present model include?
41. Lines 686-690: If the chosen values are physiologically plausible, give references and state what parts of the brain the model can plausibly represent. Based on that, the paragraph can be rearranged or rephrased to be less apologetic– using these values is a strength, and the model’s supposed correspondence to physiology can be the outset; then, mention that this choice endows TA with a much stronger effect on STD. On top of that, it can be mentioned that under other conditions the model is likely to generate comparable levels of DD with either mechanism (what conditions? Do they make for a different network, or are these different conditions of stimulation of the same network?).
42. Lines 698-699: Can the authors give a few examples for such architectures and stimuli?
43. Lines 708-711: What kind of sensitivity is referred to here, and why does the sensitivity appear to be less than would be expected in this model? On what is the expectation based? And is there a way to test how this model performs with spontaneous activity?
44. Line 722: What specific subcortical stations are the authors referring to? Wouldn’t those show DD for stimuli with shorter time intervals between them, a presentation that was not tested with this model?

**Minor Comments:**

45. Lines 43-44: What is the difference between statistically surprising sensory inputs and violations of established regularities? Aren’t such violations statistically surprising? Is it meant here that deviance detection is the ability to identify these two things or the ability to distinguish between them? Or are “violations of… “ meant as an alternative description of the previous term (in which case, it would be better to use a comma between them).
46. Line 83: Here, and in other places, the authors use the term “true deviance detection”, while throughout the work they refer to DD, without the adjective “true”. Perhaps it can be clarified that these two terms are used here to refer to the same phenomenon or that “true” is being left out for convenience.
47. Line 142 and throughout the manuscript: I suggest denoting the units of R as spikes (but leave it to the choice of the authors).
48. Line 203: Is the response calculated over the whole trial, including the interval between trials, or over a shorter time period?
49. Line 216: This sentence is not clear. Perhaps it was meant that the response “developed earlier and was very slightly larger”?
50. Line 311: in “responded more to…” – more than what? Please clarify.
51. Line 320: “higher target response” – higher than what? Please clarify.
52. Line 329: It would be more clear to replace “in reverse” with a more specific description, or explain it with another clause or in parentheses.
53. Line 344: Does “blue” refer to only some of the neurons in the right plot? If so, please clarify.
54. Line 411: Please consider rephrasing “differing by 94%” to make this clearer (e.g., X higher than Y by 94%).
55. Lines 414-415: Please be more specific (“increased local suppression” referring to the dev sequence, and “greater target responses” being a comparison to those in msc).
56. Line 421: Was the effect indeed limited to those trials, or was it just much stronger there?
57. Lines 422-424: As mentioned before, these statements are valid only for the present model and stimulation parameters, as it was already shown that networks with only STD are able to generate DD.
58. Lines 428-429: This sentence is overly complicated (“been able to lead to a build-up”), please rephrase.
59. Line 431: Consider using “the consecutive” or something similar instead of “later”.
60. Line 434: It’s simpler and clearer to refer to decay of adaptation as recovery.
61. Line 538: What is meant by “varied stimulation paradigm”? Please clarify or use a different term.
62. Line 689: What is meant by “a more even tuning”? Please clarify.
63. Line 693: The reference to Fig. 10 is not in the regular format.

**Comments on the Figures and Presentation:**

General:

a) I would advise to increase the size of text in all the figures, including axis labels and legends. Especially, in the sequence names along the y-axis in Fig. 2A.
b) Please consider using a different color for msc rather than blue (perhaps a gold, or green if its brightness is different enough from the red’s). Blue would best be reserved for the standard, as in the literature, blue and red are used for the standard and deviant stimuli, respectively.
c) In general, the rainbow-like ‘jet’ colormap used throughout is considered less friendly for color-blind readers. Please consider replacing it with an alternative one (e.g. parula, but there are other colormaps that answer the criterion as well).
d) In many of the figures there are gray lines marking the 0 (or 1, eg. Fig. 2C). Could these lines be made a little less faint?
e) In general, where the image is not an actual coordinate system (e.g. Fig. 5A-B), please give its title with an upright text, not inclined by 90 degrees like an axis label, as that is easier to read.

Specific:

a) Figure 1A: I believe the axis label of the bottom panel should have a capital V.
b) Figure 1C and 1D: Please use for the sites and stimuli colors or markings that have more contrast between them. The pale colors that were chosen are a bit difficult to distinguish and locate on the already colorful network map for people who have a common form of color blindness. Also, why do the colors of the sites and stimuli appear in different orders in the legends of 1C and 1D? 4 of the colors of 1D seem to resemble the colors of sites in 1C. Can these legends be sorted to have the same order? Or is there no correspondence between the sites of 1C and stimuli of 1D? If there is a correspondence, perhaps the authors could use the same fifth color in both? And if there is no correspondence, please use different colors in 1C and 1D (I know it’s not easy to find several distinct color palettes – here, these include the palette of the colormap used for the single neurons; if that is a problem, then for the stimuli, or for the neurons, one could use different shades of the same color).
c) Fig. 2: In the last line of the legend, the equation number is missing.
d) Fig. 3B: The asterisks marking the significance are too similar, at least in size, to the outliers. Perhaps they could be made larger or bolder.
e) Fig 4: Why are the inhibitory neurons also shown interleaved with the excitatory ones within the colormaps of this figure? Would it make sense to separate the lines in each block to an excitatory and inhibitory block?
f) Fig. 4D: What is the reason for the bimodal or multimodal distribution of ΔR?
g) Fig. 4E: Here and elsewhere, the legend has “[x variable] plotted against [y variable]”; it might be more appropriate to reverse the order to have “[y] plotted against [x]”.
h) Fig. 6: Please consider switching the axes to reflect the fact that ΔR is the dependent variable and ΔΤ the independent variable
i) Fig. 6B: In the legend, “confidence intervals” are mentioned without the percentage (which was mentioned in other occurrences throughout the manuscript).
j) Fig. 6C: What is the reason for calling ΔR a “contrast” rather than simply a difference?
k) Fig. 7, C, E, and G: Please consider switching the axes, as it would make more sense to place the quantities of the TA-only model ad the independent variable and those of the STD+TA model as the dependent variable.
l) Fig. 9, legend: In “same data plotted as lines”, consider using “curves” instead of “lines”; for “leaving 120 data points to be summarized…”, consider replacing “leaving” with a different word (e.g “providing”).
m) Fig. 10: A summarizing figure is definitely appreciated, but this figure is graphically confusing. It’s not clear why the arrows have curved shapes and where they are supposed to point or lead to – both the large arrows and the small ones, which are perhaps too small and thin, and it is not clear why they need to be curved (except for the ones forming a circle in the right-hand panels); having text on top of the arrows is somewhat of a visual load, as are the multiple formats of text that appear here (regular and bold, and different color highlights); and the general layout of text scattered at different positions is also confusing, as it is not clear why these positions are chosen and what they should mean. Some of this confusion is caused by the mixing of a topographical layout with the representation of the abstract, causal relationship between different phenomena. Again, it is excellent to have such a figure closing the manuscript – but the material it is meant to present is unarguably complex. I suggest reaching out to the university’s graphic design services or to an external graphical designer for expert advice or commission of an improved design.
