# Supplementary material for: Short-term neuronal and synaptic plasticity act in synergy for deviance detection in spiking networks
Source: PLoS Comput Biol. 2023 Oct 13;19(10):e1011554. doi: 10.1371/journal.pcbi.1011554 (PMC10599548; doi:10.1371/journal.pcbi.1011554)
Supplement: Attachment — Submitted filename: PLoS-response.docx [file pcbi.1011554.s010.docx]

Dear Editors,

We would like to thank the reviewers for their insightful, extensive, and detailed comments. We have made many alterations in direct response to these comments, including new (Fig 10) and replacement (Fig4F-H) analyses and many textual clarifications and additions. Below, we respond to all reviewer comments point by point, as is customary, copying substantially changed or added text passages from the revised manuscript as necessary.

We submit, alongside this response, a document with tracked changes corresponding to the reviewer comment responses as detailed below, retaining the format of the initial submission for ease of comparison. This document only contains the new figures; please refer to the original submission for the previous version of the figures.

Further, we submit the clean revision, now cast into the journal template. A small number of minor changes were introduced here:

- The number format in statistical reports was changed from the form “1.23e-45” to the form “1.23 x 10^-45^”, and a few floating point numbers were changed to scientific notation of the same form while preserving all presented digits.
- The referencing style (including literature, equation, and figure references) was aligned with the journal guidelines.
- We have replaced two references that previously pointed to preprints with their published counterparts (Auksztulewicz et al., 2022; and Wu and Gjorgjieva, 2022; both previously cited as 2022 preprints, now cited as their 2023 published counterparts).
- We have replaced two mentions of “data not shown” with supplementary figures (S7 Fig referring to Fig 6A, and S8 Fig referring to Fig 9A). These are mentioned in relevant comments below, but not included in the tracked document.

We hope these changes are satisfactory to reviewers and editors alike.

Kind regards,

Felix B. Kern, on behalf of the authors.

Reviewer #1: the authors use two synaptic mechanisms -- fast short term depression (STD) and slow threshold adaptation (TA) -- to explain deviance detection (DD). The consideration of TA is indeed novel and the supralinear effect of STD and TA is an interesting result worth publishing. I have major concerns which will require substantial rewriting. However, I believe these changes are straightforward to address and will significantly improve the paper.

Primary comments:

-Kern and Chao appear to use DD and stimulus-specific adaptation (SSA) interchangeably throughout the paper, when they should not. SSA is considered a neural correlate of DD but very much distinct from DD. See https://doi.org/10.1007/s00422-014-0585-7 (which I see is a paper the authors have referenced but perhaps have not read carefully).

It appears that stimulus-specific adaptation (SSA) would be appropriate for Kern and Chao to use because the ability of the network to generalize its deviance detection outside of the oddball stimulus is not tested or considered. Thus, DD should be replaced with SSA throughout the entire paper. One exception would be where a reference specifically mentions DD. If a paper mentions DD, it should be made separate from discussions about SSA. Similarly, if a paper only mentions SSA, it should not be included in a discussion about DD. One example includes a reference on line 590 for Abolafia et al 2011., where the paper explicitly mentions SSA and not DD.

-line 56: Studies of DD must carefully distinguish between repetition suppression and true deviance detection (Ross and Hamm, 2020). While this is true, the reference clearly mentions that SSA and DD are distinct and some ways they can overlap in particular experiments. The remainder of this paragraph goes on to distinguish DD and repetition suppression and correctly uses the definition of DD, but I am not sure if the authors understand that SSA is not simply repetition suppression. DD is defined according to statistics because the stimuli in DD can be far less regular than the oddball stimulus (the oddball stimulus has a repeated, regular train of tones with an occasional oddball tone that still follows the same time interval. In contrast, true DD would be able to adapt to irregular but statistically uniform sounds and detect an occasional tone that does not fit the statistics of the "usual" sounds.

As far as we understand the field, there are (at least) two ways of interpreting and using the terms DD and SSA.

1. In one interpretation, the two occupy different conceptual levels, following Marr’s hierarchy; DD is the computational goal (find and highlight deviant stimuli), while SSA is an algorithm that helps achieve this goal (adapt to frequent stimuli). We believe that Nelken (2014), referenced by Reviewer 1 above, uses this interpretation, although without an explicit definition. Following this interpretation, our use of DD, rather than SSA, cuts out the algorithmic center of the Marr sandwich, directly linking implementation (TA) to computation (DD). While this is perhaps not ideal and, given that SSA is at best a partial description of DD as shown in Nelken (2014), possibly slightly misleading at first glance, we believe referring instead to SSA would cause other problems. In particular...
2. In a second interpretation, DD and SSA are equivalent, but opposite effects. Here, one compares, as we do, the responses to a stimulus in the frequent (std) and deviant (dev) conditions to a suitable control (msc), and identifies DD as the greater response to deviant presentation (dev-msc, the deviance detection index we use), and SSA as the smaller response to frequent presentation (msc-std). This is made very explicit in Ross and Hamm (2020), and similar definitions for SSA are used e.g. in Parras et al. (2017) and in Fishman and Steinschneider (J Neurosci 2012). Our use of the term DD aligns precisely with this interpretation, and calling it SSA instead would likely confuse readers familiar with this second interpretation.

While it is highly likely that our model, in its present state, could indeed not account for more complex forms of deviance, and while of course it is a model of “only” activity-dependent adaptation, we do not agree that this makes it categorically not a model of DD. Indeed, we follow in the footsteps of many previous authors in calling the response difference between deviant and many-standards control “deviance detection”.

We concede that our use of the term DD is somewhat inconsistent: We use interpretation 1 in parts of the introduction and discussion, and interpretation 2 everywhere else. We have attempted to disentangle these notions by calling DD (per interpretation 2 above) “true DD” in the introduction and discussion, as introduced on line 61. This is in line with Ross and Hamm’s (2020) “true deviance detection” and Fishman and Steinschneider’s (2012) “genuine deviance detection”. To clarify that we are not talking about SSA (also per interpretation 2), we have adjusted line 56f to read:

Studies of DD must carefully distinguish between stimulus-specific adaptation (SSA) and true deviance detection (Ross and Hamm, 2020).

We replaced similar references to repetition suppression on lines 78 and 622 with SSA for consistency.

We have also added to the end of this paragraph a disambiguation of the two interpretations:

In the remainder of this paper, the term “true DD” (Ross and Hamm, 2020; Nelken, 2014; Fishman and Steinschneider, 2012) is used to refer to carefully controlled results, where stimulus-specific adaptation is excluded by experimental design. By contrast, unmarked “DD” is used to refer to the broader concept of deviance detection in the sense of a computational goal, which can include both SSA and true DD.

Finally, we ensured consistency with this by marking instances of DD (where it aligns with interpretation 2) throughout the manuscript.

As for references, Abolafia et al. (2011) is mentioned only with respect to threshold adaptation, rather than SSA or DD; the sentence on line 590 does not imply that the cited work touches on DD directly, but merely that neurons in auditory cortex exhibit intrinsic adaptation, as shown in their in vitro results.

-Another important note is that mismatch negativity (MMN), DD, and SSA, are distinct phenomena. Kern and Chao should again be careful about references that refer to MMN, DD, and/or SSA. I leave it to the authors to clearly establish the difference between these three phenomena and rewrite the introduction. From here on I will assume the authors use SSA in place of DD.

We understand the MMN to be a measurement of processes related to DD in the sense of interpretation 1 above, and cite references to MMN works in this sense. We are well aware that both our model and most neuron-level measurements of context-dependent differential activation in the oddball literature (SSA, true DD, or otherwise) are, at best, indirectly related to the MMN, which is why we make no direct comparisons to MMN results.

-Line 64: While many existing computational models of SSA do indeed place a significant focus on synaptic plasticity as the causal mechanism, an extremely important mechanism involves lateral traveling waves. This is a mechanism that Yarden and Nelken (2017) heavily rely on to generate SSA (in addition to population spikes, but the population spikes are closely related to the traveling wave). Indeed, Kern and Chao's network possibly contains a wave-like mechanism as noted on line 238, where there are neurons that respond earlier and later as the stimulus response propagates through the network.

The use of lateral propagation of activity in many predecessor models is mentioned in the Discussion, line 615ff, but we agree that the introduction does not do enough to establish the structural properties of previous models. We have ameliorated the paragraph in question as follows:

Existing computational models of DD place their primary focus on synaptic plasticity as the causal mechanism, acting either along feed-forward pathways to reduce transmission, or in recurrent connections to dampen the lateral propagation of activity.

-At a glance, the remainder of the paper appears to be sound, with obvious great care put into the design of the model and metrics. I will look more closely at the results once the authors address my primary concerns above, but my impression is that the paper is quite strong.

Secondary comments:

-Could the authors please comment on the choice of circular domain? If it is justified somewhere in the paper, it would be good to include it in the paragraph on line 113.

This was indeed ill motivated in the manuscript. We have added to the final paragraph of the introduction, which we hope provides the correct framing without leading readers to the premature conclusion that the manuscript is “only” about cell culture:

Neural systems that support true DD include mammalian cortical and subcortical brain areas (Carbajal and Malmierca, 2018; Parras et al., 2017; Grimm and Escera, 2012). Perhaps the simplest system known to exhibit true DD, however, is neuronal cell culture, which has recently been probed with the established paradigm for true DD (Kubota et al., 2021). Here, we take inspiration from the structural simplicity of this system, and approach true DD using a network model of spiking neurons […]

We have also added to the first sentence on line 113:

800 excitatory and 200 inhibitory neurons were placed randomly in a two-dimensional circular space with a radius of 4 mm in rough analogy to neuronal cell culture.

Finally, later in the same paragraph, we now offer justification for structure and stimulus paradigm alike:

While intentionally not an exact reproduction of the situation in cell culture – we aim to model a generic phenomenon, not a particular system – the network architecture and stimulus delivery paradigm crucially omit certain features (e.g., structural complexity, tuning curves, or adaptation in the input stimulus) that appear to be unnecessary to achieve true DD.

Reviewer #2: In this study, the authors demonstrated the important role of neuronal adaptation (implemented by threshold adaptation, TA) in supporting true deviance detection (DD), which was usually ignored or rejected in other modeling studies (e.g., Mill et al., 2011; Yarden and Nelken, 2017). This main message is well elucidated through meticulous thinking characterized by careful consideration of all relevant factors. The author also brought up the countereffect on the adaptation mechanism by inhibition (either via lateral inhibition or top-down prediction) in the discussion, which I think is worth mentioning, as the direct relationship between adaptation-based and prediction-based mechanisms is not often addressed in the studies of DD. This work would benefit future studies that consider the possibility of various forms of plasticity in sensory processing. I support the eventual publication of this manuscript in Plos Computational Biology. Most of my comments focus on clarity and readability.

Comments:

(-----clarity-----)

(1)The naming of {’dev’, ‘std’} and {’target’, ‘non-target’} is a bit confusing in the first place (even though it is described in lines129-136) because usually ‘dev’ means the ‘target’ rather than a condition. A re-consideration of the naming may improve the clarity.

We considered this carefully and came to the conclusion that a renaming is warranted. Unfortunately, there does not seem to be a consensus on how to name these sequences (particularly what we called ‘std’). Thus, to make the situation as clear as possible, we have chosen to name the sequences “A-as-std”, “A-as-dev”, and “A-in-con” throughout the manuscript, shortening to “A:std” etc. in figures where the full name would be distractingly long. However, note that the previous sequence naming is maintained in the present document. We hope this is sufficiently clear, but remain open to better suggestions.

(2)The mechanism of TA in DD is well described (in Figures 4, 5, and 6) and is convincing. This may also account for a related experimental observation, where the amplitude of a deviance response depends on the distance between the ‘dev’ and ‘std’ tones: A larger distance elicits a larger deviance response. This phenomenon is reproduced by an adaptation-based model (e.g., Fig. 2B in May and Tiitinen, 2010). The authors mentioned using different pairs of sites for A/B tones (lines 137-138). Could the authors comment on whether their model reproduces such a phenomenon?

We added a small investigation into this, and added it as panel A to a new figure 10, along with an explanatory paragraph:

We tested two ways in which the stimulus paradigm might influence the expression of true DD. Firstly, since our data contained both nearby stimulus pairs (2.9 mm apart) and distant pairs (4.8 mm apart), it is reasonable to ask whether the nearby pairs were more affected by local fatigue. As shown in Figure 10A, these two groups did not differ in their average deviance detection index (t[118] = 0.71, p = 0.479, independent samples t-test), indicating that greater proximity between stimulus locations in the oddball sequences did not degrade the deviance detection capability in general. We note that, unlike e.g. in auditory cortex, where a particular stimulus evokes responses only in a small part of the network, the situation here is that a majority of neurons responds to any given stimulus, that is, the receptive fields of the neurons overlap to a very large degree, which may explain the absence of a distance dependence.

We did find, however, that the DDI varied more among nearby stimulus pairs (F[2,60] = 9.92, p = 0.00208, Brown-Forsythe test for equality of variances). Without investigating this in detail, we suspect that while the greater proximity may have led to an overlap in early-responding populations, the overlap may have been sparse enough to yield both smaller deviant responses in some cases, through fatigue of excitatory pathways, and larger deviant responses in others, through fatigue of isolated inhibitory pathways. We further note that the cell culture data in (Kubota et al., 2021), Figure 4, show a similar trend towards greater variability in overlapping stimulus pairs.

(3)Line 141: I am not certain what the 4 data sets are. Could the authors make it clearer?

The awkward formulation is an unfortunate side effect of trying to conserve and re-use as much data as possible. We have reformulated the opening sentence of the paragraph as follows:

In each network, we ran 4 oddball sequences, using the following stimulus site pairings as target/non-target (A/B) pairs: 1/2, 2/1, 3/5, and 5/3, yielding both dev and std sequences for all 4 stimulus sites involved. In addition, we ran one msc sequence involving all stimulus sites, the results of which were shared between the data sets from one network.

We hope this makes the situation clearer.

(4)Line 197: The authors mention the second peak (at around 20 ms) in the std condition. The second peak only happens in the STD+TA condition. This seems to be not observed in reality. Could the authors comment on this?

At that point in the manuscript, we are merely describing what we see, and it would be an error of omission not to mention this dynamic. Spot checks among networks other than the sample we used does not yield a pattern of this kind; indeed, the temporal evolution of the spike response varies a lot between data sets.

(5)The authors use a simple model (without too many constraints and fine-tuning of parameters) so that the underlying mechanisms of DD can be elucidated more clearly. It would be good to add more explanation about the simplification and the corresponding effect. For example, (a) the decision to present stimuli instantaneously rather than over a period (e.g., 50 ms), (b) the decision to arrange the five sites circularly rather than linearly.

These choices were coherent, but ill motivated in the manuscript. We have added to the final paragraph of the introduction, which we hope provides the correct framing without leading readers to the premature conclusion that the manuscript is “only” about cell culture:

Neural systems that support true DD include mammalian cortical and subcortical brain areas (Carbajal and Malmierca, 2018; Parras et al., 2017; Grimm and Escera, 2012). Perhaps the simplest system known to exhibit true DD, however, is neuronal cell culture, which has recently been probed with the established paradigm for true DD (Kubota et al., 2021). Here, we take inspiration from the structural simplicity of this system, and approach true DD using a network model of spiking neurons […]

We have also added to the first sentence on line 113:

800 excitatory and 200 inhibitory neurons were placed randomly in a two-dimensional circular space with a radius of 4 mm in rough analogy to neuronal cell culture.

Finally, later in the same paragraph, we now offer justification for structure and stimulus paradigm alike:

While intentionally not an exact reproduction of the situation in cell culture – we aim to model a generic phenomenon, not a particular system – the network architecture and stimulus delivery paradigm crucially omit certain features (e.g., structural complexity, tuning curves, or adaptation in the input stimulus) that appear to be unnecessary to achieve true DD.

(6)The result in this work shows no significant DDI that differs from 0 in the STD-only condition (line 222). Is it due to the instantaneous stimuli or other configurations (e.g., time constant, SOA) that differ from other modeling works emphasizing STD?

This is largely a consequence of the short time constant of STD (150 ms) compared to the SOA (500 ms). We have added a short investigation into the effect of SOA in a new figure (10B), with accompanying text:

[… W]e investigated the impact of stimulus onset asynchrony (SOA). The apparent inability of STD to induce a significant DDI (see Figure 3) stands in contrast to the preceding literature, where STD, not TA, was used successfully to model deviance detection (Mill et al., 2011; May et al., 2015; Yarden and Nelken, 2017). Given that the stimulus onset asynchrony (SOA, 500 ms) was much greater than the STD time constant (150 ms), but shorter than the TA time constant (1 s), it seemed likely that this discrepancy was involved in exaggerating the effect of TA relative to STD in our analysis. To confirm this, we systematically manipulated the SOA and quantified the DDI of the model with either one or both of the plasticity mechanisms.

As shown in Figure 10B, we find that STD is entirely capable of inducing true DD by itself at an SOA of 200 ms and below. By contrast, the DDI of the TA-only model becomes moderately larger with shorter onset asynchronies, but shows signs of saturation and is surpassed by the STD-derived DDI at an SOA of 50 ms. Neither mechanism induces any significant DDI at a long SOA of 1 s. In addition, we find that the synergy effect, where the DDI of the full model significantly exceeds the sum of the DDIs of the two mechanisms individually, extended over onset asynchronies of 350, 500, and 750 ms. However, at shorter SOAs, we instead find that the full model DDI was smaller than the sum of the ablated model DDIs, which we tentatively interpret as due to saturation.

(7)In line 426, 0.0013 is calculated using 1s in the numerator, why not 500ms (the SOA)?

The point we are trying to make here is that, although there may be depression carried over between successive trials (line 421), such carry-over does not extend over two trial intervals, such that the “memory” imbued into the network by STD cannot reliably accumulate and is limited to a single trial.

(8)Line 611: A DDI map of two factors (e.g., \tau_{STD} and \tau_{TA} within a reasonable range) may help with the robustness in the parameter space.

That may be worth doing in a subsequent study, particularly with an eye towards comparing the model’s behavior to physiological data, but we feel that such an investigation is beyond the scope of the current work.

(9)In line 621, providing a brief description of the predominant thinking will help readability.

We have added the following brief explanation:

In contrast to the predominant thinking in the field (see e.g. Ross and Hamm (2020) for an extensive review), which suggests that active cancellation may be required for true DD, our work suggests [...]

(-----Accuracy, Scientific formality-----)

(10)In Equation 1, a resistance variable R on the right-hand side is missing. Usually, it should be something like [V_{rest}-V_m] +R*I_{syn} so that they are in the same unit. Need some clarifying text if only a variable I_{syn} is used here.

The numerical integration was indeed done with unit-less g_e, g_i, and w, and thus I_syn in volts rather than amps. Our intention was not to provide a biophysically highly accurate model, so we were interested in avoiding having to specify parameters we deemed of little direct relevance to our questions. However, this appears easy enough to fix assuming R_m = 100 MOhm and w = 0.1nS, which seem to be acceptable ballpark figures. We have adjusted Equation 1 and definitions of w accordingly.

(11)In line 93, the expression “with = 30 ms the membrane time constant and = −60 mV the resting membrane potential” can be written as “with the membrane time constant = 30 ms and the resting membrane potential = −60 mV”. The rest of the contents have the same problem.

We have adjusted the parameter descriptions to equations 1-3 as suggested.

(12)Line 155: “...with A and B in the msc sequence and calculated z-scores with respect to the population” is not clear to me. Maybe some equations would help?

The situation is not so complex (or important) as to call for an equation, but the wording was indeed rather unhelpful. We have revised the description of the selection process as follows:

The sample network was chosen to be roughly representative of the analyzed data sets as follows: In each data set, we assessed the average number of spikes fired in response to stimulation with A and B in the msc sequence. We considered only data sets whose response size for both A and B was within one standard deviation of the mean response size across data sets, then hand-picked a sample data set (a network and stimulus assignment, hereafter referred to as the “sample network”) that exhibited both an increased deviance detection index (see Equation 12 below) in STD+TA over TA only, and greater msc than std responses in both STD+TA and TA only conditions.

(13)Line 189: Measurements such as equation 6 can be described in the Method section (and maybe also equations 7 and 8)

We moved Eqn 7-8 and the corresponding explanations into the Methods:Notation section, which we accordingly renamed “Notation and quantities”, adjusted the text, and aligned the stimulus variable (“X” in the pre-existing Notation text, “S” around Eqn 7-8) to “S”.

We also moved the technical paragraph preceding Eqn 6 into the same section.

(14)Lines 221-225: For comparisons of STD with 0, the authors employed a two-tailed Wilcoxon test, considering the possibility of either an increase or decrease in values. Conversely, for comparisons of TA with 0, a one-tailed Wilcoxon test was used. Please provide the reason(s); otherwise, the statistical method (one-tailed or two-tailed) should remain consistent. However, the authors did not explicitly state the comparison method when assessing the full model against 0. This ambiguity exists throughout the whole paper. I recommend providing the name of the statistical method used for this comparison to ensure consistency in statistical analyses.

We have adjusted the comparison for the STD-only DDI to be one-tailed (>0) like the other tests. Our reasoning for a two-tailed comparison was that, since the median is negative, readers might draw the wrong conclusion from a one-tailed test for positivity, but consider that to be lower priority, on the whole, than consistency between tests.

Since we frequently report the results of several related tests, we chose to omit, within paragraphs, any test parameters (test type, n, etc.) that are repeated. We believe this aids overall readability while still presenting the necessary details.

(15)The statistical parameters (e.g., p-value p) should be italicized.

Interestingly, the journal submission guidelines say nothing to the effect; however, we see no reason not to adhere to APA standards and have adjusted typography accordingly.

(16)The Wilcoxon statistic results are typically stated in terms of Z-score rather than T-score.

We agree that Z-scores are more meaningful, and have replaced Wilcoxon T-scores with Z-scores where applicable. As a result, the disambiguation on lines 175f became unnecessary and was removed.

(-----Figures-----)

(17)Figure 1: The colors for Exc and Inh in Fig.1A and Fig. 1B are not consistent. The color contrast for sites 1-5 in Fig.1C is too low, which makes it hard to identify them in the network. Marking sites 1-5 directly on the graph may help with clarity.

We have aligned exc/inh colors in panels A and B, and replaced colors with direct labeling of stimulus locations in panel C.

(18)Figure 3: Panel C is mentioned in the legend but missing in the figure.

Fixed, this was meant to be a reference to panel B.

(19)Figure 4 Legend: Description in F, G, and H is difficult to understand.

In retrospect, not only the description, but also the content of what was shown in these panels was difficult to understand. We have revised the analysis leading to these panels, resulting in a clearer picture of the effect we were trying to show without substantially changing the point we were hoping to make.

To increase clarity, we have chosen to describe the methods applied in Figure 4 in a subsection (“Contribution of TA to greater activity”) of the “Notation and quantities” part of the Methods, which we refrain from reproducing here due to its length; please see the revised submission.

Naturally, the accompanying paragraph in the main text, previously on lines 279-292, was also changed substantially:

Yet, as we saw in Figure 4C, the membrane voltages also differed between the two sequences. Since firing is defined as the membrane voltage crossing the firing threshold, differences in either of these two factors could explain differences in activity. Therefore, considering instances of greater activity in dev than msc, we wanted to know to what extent these differences were caused by lower thresholds, rather than higher membrane voltages. To this end, we calculated the relative positive contributions of 𝜃 and 𝑉_𝑚_ to instances (i.e., histogram bins) with greater activity as described in Methods. The resulting histogram is shown in Figure 4F. We see that in most bins, particularly along the response front, the major contribution was provided by 𝑉_𝑚_, as indicated by the blue shading, while greater activity after the main response, as well as during its very early portion, were driven largely by lower 𝜃. A similar picture emerges when controlling for the magnitude of the activity difference and averaging across neurons, as shown in Figure 4G, and is maintained across datasets, as shown in Figure 4H. This indicates that adaptation initiated and dominated the stronger firing at the start of trials, whereas the later response was enlarged primarily as a result of greater presynaptic excitation. In short, lower thresholds kickstarted a self-reinforcing burst of activity.

(20)Figure 5: Can add A and B circles on all subplots for clarity.

We have added location markers to all subplots in all relevant figures (1, 5, 6, and 8). In order to avoid biasing the appearance of color inside vs outside the marked locations, we also changed the markers to small foreground stars rather than large background circles.

(-----Proofreading-----)

(21)Lines 139-140: (labeled B to E) or (labeled “B” to “E”)? need to be consistent here.

Fixed.

(22)Figure 2 legend: “DDI, see Equation eq:ddi” A link to the equation is missing.

Fixed.

(23)Line 153: 120 networks or 120 data sets?

We meant 120 (networks and stimulus pairs). This appeared often enough to warrant a re-examination; we have changed the formulation to “data sets” instead, both here and elsewhere.

(24)Line 221: Figure 3C or Figure 3B?

Fixed, see also comment (18).

(25)Line 275: “during oddball sequences”, or more specifically “during dev sequences”?

By design, every dev sequence for one target stimulus is the std sequence for its non-target partner. In addition, since T^A^ is measured at the start of target trials, before any activity propagation occurs, and since target trials occur at random intervals within the sequence, T^A^ ~= T^B^. Thus, the statement applies to both std and dev sequences.

(26)Proofreading may be needed. I assume I didn’t find all of them.

While we all strive for perfection, it is typically expedient to stop somewhere along the asymptote.

Reviewer #3:

This modeling work By Kern and Chao presents an investigation of possible mechanisms for the neuronal process of deviance detection, which is an important building block of sensory processing and scene analysis, and has been studied extensively in the context of human cognition, as well as in animal models.

This work introduces into the field of modeling deviance detection and related phenomena the well-known neuronal property of threshold adaptation. The authors argue correctly that this inherent neuronal process has so far been left out of theoretical studies, even though it is obviously present in the systems being investigated, and as a dynamic adaptation process is likely to have some involvement in the way neuronal systems adapt to frequent and regular stimulation.

The progress presented in this work is not conceptually innovative, as it takes the logical next step that is needed in the development of its field. However, there is merit in the authors’ choice of model and in their construction of the neural network that they used. They elegantly apply a network of integrate-and-fire neurons, which allows an explicit inclusion of both threshold adaptation and synaptic depression as well as access to the dynamics of single spikes, while avoiding details that are probably unnecessary for investigation at the level of network responses. Furthermore, they employ a simple connectivity scheme and avoid an explicit description of any specific stimulus space, sensory system, or brain area, demonstrating the phenomenon on the general, conceptual level and extracting principles that are likely generalizable to different occurrences of deviant detection in the brain.

The investigation presented in this manuscript is well conducted, delving into the complexity of having two mechanisms with similar dynamics and outcomes performing side-by-side and in synergy, and providing rigorous evidence for its claims. It produces a solid demonstration that threshold adaptation should be included in models set on investigating deviance detection, and of its workings and impact.

My recommendation is that this manuscript be accepted pending minor revisions. The general points of which I am critical are that this work does too little to support its choices of network structure and parameters on physiological evidence from real neuronal networks operating in the brain that show deviance detection, and that it does not do enough to relate its findings to the actual phenomenology found in experiments (e.g. in the strength of deviance detection and the temporal profile of responses to truly deviant vs. infrequent stimuli). In addition, there is insufficient investigation of the impact of choices that were made in constructing the model, or justification for not investigating it. As a result, the authors’ claim that threshold adaptation is a major driver of deviance detection, with synaptic depression acting to support and enhance it, is put in doubt, as it is not clear how much this claim is specific to the way the network was constructed or to the stimulation paradigm that it was presented with. An acceptable solution to this would include either of the following: (a) simulations where the network is stimulated at different frequencies, with an investigation of the resulting dynamics to demonstrate whether the dominance of threshold adaptation applies also for higher-frequency stimulation; or (b) grounding the choices of network parameters and connectivity in evidence from the experimental literature, to show that the brain area(s) being studied, when modeled with reasonably close parameters and stimulated similarly to experimental studies, are plausibly relying on threshold adaptation more than on synaptic depression for their deviance detection.

The point of our manuscript is absolutely not to diminish the importance of synaptic depression in this context, and we agree that the manuscript does not do enough to emphasize this. Our intention is to show that TA can lead to DD, not that STD cannot; it is only through unfortunate choice of parametrization that the results turned out as extremely lopsided as they did, and we failed to rectify this at a sufficiently early stage in the project because we were focused primarily on TA. As an immediate remedy along the lines of the requested (a), we have added a brief investigation into the dependence of the deviance detection index on the stimulus onset asynchrony, and present in a new Figure 10 (B), along with the following discussion:

[… W]e investigated the impact of stimulus onset asynchrony (SOA). The apparent inability of STD to induce a significant DDI (see Figure 3) stands in contrast to the preceding literature, where STD, not TA, was used successfully to model deviance detection (Mill et al., 2011; May et al., 2015; Yarden and Nelken, 2017). Given that the stimulus onset asynchrony (SOA, 500 ms) was much greater than the STD time constant (150 ms), but shorter than the TA time constant (1 s), it seemed likely that this discrepancy was involved in exaggerating the effect of TA relative to STD in our analysis. To confirm this, we systematically manipulated the SOA and quantified the DDI of the model with either one or both of the plasticity mechanisms.

As shown in Figure 10B, we find that STD is entirely capable of inducing true DD by itself at an SOA of 200 ms and below. By contrast, the DDI of the TA-only model becomes moderately larger with shorter onset asynchronies, but shows signs of saturation and is surpassed by the STD-derived DDI at an SOA of 50 ms. Neither mechanism induces any significant DDI at a long SOA of 1 s. In addition, we find that the synergy effect, where the DDI of the full model significantly exceeds the sum of the DDIs of the two mechanisms individually, extended over onset asynchronies of 350, 500, and 750 ms. However, at shorter SOAs, we instead find that the full model DDI was smaller than the sum of the ablated model DDIs, which we tentatively interpret as due to saturation.

We further qualified the opening line to the investigation into the role of STD (line 377f) with the trial interval used for additional clarity:

As noted previously, STD alone exerted no true DD effect at a trial onset asynchrony of 500 ms, but its addition did enhance the true DD effect established by TA.

I have listed in my comments additional doubts and questions that I had about the operation of the model with different choices of parameters, e.g. of connectivity and of inhibition. While I would appreciate consideration of these and possibly simulations to address them, I would not require full investigation of those points and characterization of their results in the present context, as they would increase the scope of the work by too much, and are possibly a matter for potential independent follow-up studies where they can be granted the focus and the thoroughness they deserve.

**Comments on the Manuscript:**

**Major Comments**

1. In the author summary, and then in lines 50-55, the authors mention that improved understanding of deviance detection could help develop treatments for several disorders. Would they be able to elaborate, or hypothesize, on how the understanding gained in the present work sheds light on the aberrant processing or the symptoms in the said disorders, and might guide the development of new treatments? (this does not need to go into the paper, unless the authors would like to mention it somewhere).

Our understanding of the nature of these disorders is that, in some way or another, predictive processes are disrupted compared to healthy subjects. Our suggestion is of a rather general nature, namely, that in TA, there is a mechanism which may be involved in such processes and which was previously not strongly considered as a possible point of failure or remedy. We readily acknowledge that it is a long way from here to clinical application, which we are not prepared to undertake in full, including steps such as confirming this involvement in physiology, linking disruptions in neuronal adaptation (e.g. defects in some of the many potentially relevant ion channels) to behavioral disorder pre-clinically, and finally targeting such defects pharmacologically.

2. In line 59, true deviance detection is described as an “enhancement” of a response in one condition compareג to another. However, the responses to true deviants are not enhanced by the regular background, but merely less reduced than responses to the same stimuli when they appear over an irregular background.

In general, throughout the manuscript the authors use descriptions like “increase”, “reduced” “reduction”, etc. in comparisons of responses and quantities that are taken from the different stimulation sequences. This can imply that there is a dynamical process that leads from one situation to the other, which is confusing. It would be best to replace this terminology with descriptions like “higher/lower than X”, “strong(er)/weak(er) than X”, etc., making sure to name both of the quantities being compared (for negative quantities, “more negative/less negative” to avoid confusion).

Examples include “increase” in line 241; “decrease” in line 277 (it’s not that T is decreased, just that it is lower/less increased in the dev sequence than in msc); “reduction” in lines 294, 316; “increasing” in lines 343, 362 and “decreased” in lines 344, 353; “increased” in lines 390, 391; multiple instances in lines 393-418; “reduction” in lines 436, 456, etc. (also line 657).

We agree with this observation. The use of these apparently active terms likely arose from a desire to use nouns, where “the fact of X *being* larger” and “the fact of X *becoming* larger” are indistinguishable. We have revised our use of these terms throughout the manuscript, in reference to response size and other quantities alike, in an effort to promote clarity over syntactic expedience.

3. Line 75 (and also 583-584): It is not accurate that TA was rejected a-priori as a mechanism for DD. Rather, TA was in a way skipped in the consideration of mechanisms for DD, since it was already rejected as a mechanism for SSA: Yarden and Nelken mention that intrinsic neuronal plasticity cannot easily explain SSA, while reviewing the reasons that models that preceded theirs have used STD and not TA. However, those previous models were all of the feed-forward type, so TA was taken out of the equation already when the field was focusing on feedforward models, and was overlooked as a potential mechanism when the architecture is of a recurrent network. The results of Yarden and Nelken demonstrated that in fact, TA should have been reconsidered: once a recurrent network is used, that has some compartmentalization, TA should support SSA and DD in much the same way that their cortical columns do by STD, since each of their columns essentially served as a unit that is mostly adapted by one stimulus (and more weakly adapted by other stimuli) – a manner of adaptation that could just as well be caused by TA. This was of course not recognized in their paper. The details of this course of development do not need to be laid out in the present manuscript, but consider refining the point about how TA was rejected or overlooked.

We have added to the relevant sentence in the introduction:

[…] rejected it a priori as not sufficiently stimulus specific, and therefore incapable of producing either SSA or true DD (Yarden and Nelken, 2017).

We still think, however, that the rejection was unwarranted: From a computational point of view and ignoring quantitative differences, whether a neuron’s output is reduced by virtue of an increased threshold or by virtue of a reduced PSP, the downstream result (reduced input) is similar. Further, to the extent that neurons themselves respond in stimulus-specific manner (i.e., are tuned to certain stimulus properties), the adaptation too is no less stimulus-specific one way or another.

4. Line 99: Why were the inhibitory synapses not made to have STD? It is known that the synapses that fast-spiking (PV) neurons make on excitatory neurons in cortex are depressing. Can the authors provide the reasoning behind not using STD in inhibitory synapses (esp. since they are explicitly modeling fast-spiking neurons)? Can they give a hypothesis about how leaving this physiological detail our of their model might have affected their results or their applicability to cortical networks (or test how they might change if this feature is added)?

Our (admittedly weak) justification for this is that we followed bad old modeling traditions and treated inhibitory neurons and synapses as largely passive units. We have added a subsection (“Limitations and future work”) where this is discussed:

Firstly, in constructing our model, we followed some rather outdated modeling practices, particularly in relation to inhibitory neurons and their synapses, which we modeled without adaptive processes. However, GABAergic neurons are known to be capable of both cellular adaptation and short-term synaptic plasticity (Perrenoud et al., 2013; Markram et al., 2004; Gupta et al., 2000). In addition, even our excitatory synapses only depress, while of course facilitating and depressing-facilitating synapses are likewise common in cortex (Tsodyks and Markram, 1997; Varela et al., 1997). It is difficult to predict how these additional plasticity mechanisms would affect our results. We may speculate at obvious immediate effects – adaptation in inhibitory neurons and facilitation in excitatory synapses would likely work to counteract the processes detailed in our work – but, as we have shown, interactions between plasticity mechanisms can be surprising and complex.

5. Line 102: How were tau_x and U chosen? If they were based on physiological data (they are indeed correct for auditory cortex, for example), please cite the sources for these values and what parts of the brain they might simulate (in another place, line 631, this model is stated to correspond to, or also to, subcortical areas – do these values apply to those areas? Would they need to be modified for that?).

We did not target a specific area, as our aim was to establish a generic working principle. U was chosen somewhat arbitrarily, given the wide range of plausible values reported in Tsodyks and Markram (1997), while tau_x was chosen based on auditory cortex data from the David lab. We have added suitable citations on line 102, and added to the discussion of differences between TA and STD:

For synaptic depression, 150 ms, chosen based on thalamocortical inputs to primary sensory areas (David and Shamma, 2013; Pennington and David, 2020), is at the lower end of the plausible range for cortical neurons, with values reported ranging from hundreds of milliseconds to several seconds for intracortical synapses (Tsodyks and Markram, 1997; Markram et al., 1998; Varela et al., 1997; Asari and Zador, 2009; Levy and Reyes, 2011).

As for line 631, we have softened the claims here ([…] our model may provide a possible explanation […]) and in the subsequent title (Adaptation may support subcortical DD) to bring them into line with the considerable uncertainty in regards to the applicability of our model to any particular brain structure.

6. Line 102+3 (there are several lines between 102 and 103): What is the reason for making all of the inhibitory neurons fast-spiking (and non-adapting)? Can the authors provide a hypothesis (or a test) about the impact of non-fast spiking neurons in this network on DD?

Given the small number of spikes fired per neuron and trial, we don’t believe that the refractory period has a strong impact, other than by altering the response of the neurons under immediate stimulation. We have added a small discussion on this and related points in the new “Limitations and future work” section:

Further, across both inhibitory and excitatory neurons, many simplifying assumptions were made for expedience, many of which (e.g. cellular, synaptic, and structural homogeneity) bear closer examination and better grounding in physiology in future work. Even within the confines of point-process LIF models, much greater biological plausibility could be achieved.

7. On a side note, the model presented here appears suitable to test the possible effect or role in DD of facilitating synapses, both between excitatory neurons (an interesting prediction is that they might actually enhance the responses to infrequent stimuli, following Mongillo et al., Science 2008), and from inhibitory to excitatory neurons (namely the facilitating synapses that Martinotti cells make on the excitatory neurons). The authors are encouraged to consider testing these things in their model, but of course that would substantially increase the scope of this work, and may be more appropriate for a different paper to follow up on this one.

We appreciate the enthusiasm, but we believe this is out of scope for the current work. We do touch upon facilitating synapses under Limitations and future work (see response to comment #4).

8. Line 103: Given that tau_theta is much longer than tau_x, it appears almost inevitable that TA would prove to have a greater effect on DD than STD. However, this also depends on the parameters of the paradigm used to stimulate the network. Would the expected relative contributions of STD and TA to DD be different with shorter intervals? Have the authors tried to run the paradigm with shorter intervals between the stimuli, or can they provide an example that demonstrates similar or different relative contributions (e.g., runs of the sample network stimulated with shorter intervals, ablated as in Fig. 3)?

We have analyzed the effect of different, particularly shorter, onset asynchronies in the new Figure 10 (see our response to (a) above). If our choice of SOA for the main analysis appears biased, it is not out of a desire to marginalize the importance of STD, but rather out of a desire to investigate an interesting result (the synergy between TA and STD).

9. Line 114: Could the authors provide an explanation or reference for the choice of having each neuron form 50 synapses, and the ranges of the different synapses? What is the resulting probability of connection and how does it relate to known connectivity patterns in the brain? What area of the brain are these parameters supposed to model (and how would they be different for other areas that the authors claim to capture with the model or its principles, like cortical/subcortical areas)? How would the authors expect different choices to affect the DD in the network?

These values were chosen manually to produce reliable responses without giving rise to instabilities, ongoing or stereotyped activity patterns, and the like. We have added a statement to this effect to the paragraph in question:

The particular values were chosen to achieve reliably large stimulus responses without giving rise to instabilities or ongoing activity.

The resulting connection probabilities within the patch the neurons project to (i.e., p(connect to target|target in range)), are 0.8 for inhibitory and 0.2 for excitatory neurons.

As for brain areas, we did not target any area in particular, but rather aimed to model a generic phenomenon with minimal assumptions (and assumption of minimality), taking inspiration from related findings in cell culture, but we had done too little to explain this to the reader. We have added to the final paragraph of the introduction, which we hope provides the correct framing without leading readers to the premature conclusion that the manuscript is “only” about cell culture:

Neural systems that support true DD include mammalian cortical and subcortical brain areas (Carbajal and Malmierca, 2018; Parras et al., 2017; Grimm and Escera, 2012). Perhaps the simplest system known to exhibit true DD, however, is neuronal cell culture, which has recently been probed with the established paradigm for true DD (Kubota et al., 2021). Here, we take inspiration from the structural simplicity of this system, and approach true DD using a network model of spiking neurons […]

We have also added to the first sentence on line 113:

800 excitatory and 200 inhibitory neurons were placed randomly in a two-dimensional circular space with a radius of 4 mm in rough analogy to neuronal cell culture.

Finally, later in the same paragraph, we now offer justification for structure and stimulus paradigm alike:

While intentionally not an exact reproduction of the situation in cell culture – we aim to model a generic phenomenon, not a particular system – the network architecture and stimulus delivery paradigm crucially omit certain features (e.g., structural complexity, tuning curves, or adaptation in the input stimulus) that appear to be unnecessary to achieve true DD.

10. Line 114: Why are all synapses chosen to have a weight w = 1? Why not a gradual decrease in weight with distance? Why are the inhibitory and excitatory synapses of the same weight? Was this chosen empirically for maintaining a specific balance (and if so, what were the criteria?), or depends on the connectivity pattern – or based on physiological data? What would be the effect on DD of choosing to have differently-weighted excitatory and inhibitory synapses, or randomly weighted synapses with a profile of the strength decaying with distance between the somata?

These (over-)simplifying choices were made for expedience, rather than in reference to a target system. We speculate that a less artificial, better balanced connectivity structure would have led to more dynamic responses, but also to a clearer picture of the spatial patterns of fatigue, particularly if weights were distance-dependent. Conversely, DD effects would likely be weaker, due to higher activity and necessary rebalancing in the plasticity parameters.

11. Line 119: How was the number of stimulated neurons chosen? And the strength of this stimulation? Is stimulating 10 neurons at 2-3 spikes each meant to model or is based on data from any specific physiological system? If so, please provide references. What would be the effect, and the consequences for DD, of having the stimulus evoke responses of different magnitudes in an area out of the network according to a tuning curve? Finally, are the stimulated neurons only excitatory neurons, or also inhibitory? Please specify in the text.

Stimulation was likewise inspired by cell culture experiments (see response to comment #9). In culture, stimulation is delivered through extracellular electrodes in a multi-electrode array, which is roughly mirrored in our setup. While it may have been more consistent to stimulate all cells within a small radius instead of a fixed number, we do not believe that this would noticeably change our results.

Inhibitory neurons were also stimulated and counted towards the set of 10; we have clarified this in the relevant paragraph.

We speculate that stimulus tuning could have increased the overlap between the responses to adjacent stimuli, giving rise to a distance dependence of DD (see response to #13 below).

12. Stimulation of this model was performed by a one-time increase in the excitatory conductance. What would be the effect on the network and on DD of a longer stimulus?

As an immediate consequence, since our model is not a balanced network, longer stimulation would directly increase activity levels, possibly to the point of instability or saturated adaptation. Balancing the network or reducing the stimulus magnitude to accommodate such a scenario would likely result in an attenuated DD effect.

13. Lines 137-138: Was there any difference in terms of DD or otherwise between the two stimulus pairs used?

Given our interpretation of local/global fatigue, we felt that this is a question worth fully addressing in the manuscript. We have added a panel (A) to the new Figure 10, along with a paragraph explaining the results:

We tested two ways in which the stimulus paradigm might influence the expression of true DD. Firstly, since our data contained both nearby stimulus pairs (2.9 mm apart) and distant pairs (4.8 mm apart), it is reasonable to ask whether the nearby pairs were more affected by local fatigue. As shown in Figure 10A, these two groups did not differ in their average deviance detection index (t[118] = 0.71, p = 0.479, independent samples t-test), indicating that greater proximity between stimulus locations in the oddball sequences did not degrade the deviance detection capability in general. We note that, unlike e.g. in auditory cortex, where a particular stimulus evokes responses only in a small part of the network, the situation here is that a majority of neurons responds to any given stimulus, that is, the receptive fields of the neurons overlap to a very large degree, which may explain the absence of a distance dependence.

We did find, however, that the DDI varied more among nearby stimulus pairs (F[2,60] = 9.92, p = 0.00208, Brown-Forsythe test for equality of variances). Without investigating this in detail, we suspect that while the greater proximity may have led to an overlap in early-responding populations, the overlap may have been sparse enough to yield both smaller deviant responses in some cases, through fatigue of excitatory pathways, and larger deviant responses in others, through fatigue of isolated inhibitory pathways. We further note that the cell culture data in (Kubota et al., 2021), Figure 4, show a similar trend towards greater variability in overlapping stimulus pairs.

14. Fig. 2: The responses shown here are averaging over the whole network. What experimental paradigms from the literature should this be compared to?

A direct comparison can be drawn to work in cell culture (Kubota et al., 2021). For in-vivo work, the closest paradigm would probably be local field potential recordings, although we are aware that those are strongly influenced by dendritic inputs, rather than somatic activity.

15. Fig. 2: What do the responses of single neurons in the network look like? Do they all have dev > std and dev > msc? Can the authors provide a distribution or a scatterplot, perhaps from the sample network?

Almost all neurons (except for a silent minority) do follow the same pattern. We have added two panels to Fig 2 and an accompanying description following line 200:

Individual neurons responded in a similar pattern, shown by way of the distribution of single-neuron response magnitudes in Figure 2C. The dev sequence targets evoked higher responses in a large portion of the network. Subtracting the dev response from either msc or std, however, we see that a bimodal distribution emerges, with some neurons showing little to no difference in their response. This second, unchanging portion consists mainly of neurons that respond very little in any sequence. Removing such unresponsive neurons (specifically, the 204 neurons that fire less than 0.1 spikes per trial in any sequence), this portion largely disappears, and the distributions become unimodal and largely positive, indicating that neurons that are sensitive to the target stimulus respond most strongly in the dev sequence.

16. Fig. 2: Do the different time-profiles found here for the dev, msc, and std responses resemble any experimental results from the literature?

We are not aware of any close correspondences, but should also point out that the time courses varied significantly between data sets:

We could readily identify similar response patterns emerging across most data sets, though details of the temporal response profile varied widely.

17. What is the significance of the finding that most networks had a positive DDI? I am presuming that the authors take this to mean that their model can generate DD without a significant dependence on the detailed connectivity. However, for establishing that TA can generate DD, wouldn’t it suffice even if only a minority of networks were able to show DD?

For a proof of existence, a minority would indeed suffice. However, this would immediately raise the question of determining factors. By showing that DD is an almost universal outcome in our model, we presume to infer that neuronal and/or synaptic fatigue are a primary mechanism that does not depend on e.g. microstructural details of the circuitry.

18. Why is STD so ineffective in generating DD in this model? Yarden and Nelken’s model had only the STD and was able to generate DD, albeit not nearly as strong as that found here (though they did not compute a DDI nor took the response over the whole network).

The obvious proximal reason, of course, is in the interaction between time constant and SOA, as demonstrated in the new analysis in Figure 10. We also note that the stimulus response – or any activity, for that matter – rarely lasted longer than 30 ms or so, both because of the point-like nature of the stimulus, and because the networks were likely over-damped. A longer response would naturally reduce the silent time between inputs, thus at least partly mending the gap between SOA and time constant.

19. Line 220: I would like to see a comparison of the DDI in this model with similar quantities from experimental studies (could be here or in the discussion section).

We have added a short paragraph to the model comparison subsection of the Discussion, which reads as follows:

Finally, a comparison between models is incomplete without mention of the magnitude of true DD quantified in the DDI. Across our datasets, DDI at an SOA of 500 ms varied substantially, with the bulk of values contained between 0 and 0.2. This is similar to experimental data from cell culture probed at the same SOA (Kubota et al., 2021). Likewise, Yarden and Nelken (2017) report in their model values around 0.2-0.3 for pairs of nearby stimulus sites, while at greater separations their DDI is well above 0.5. Of the model variants in Mill et al. (2011), the ABC variant, which is most similar to ours and was fitted to experimental data (von der Behrens et al., 2009), shows substantially larger values around 0.4, though also at only half the deviant occurrence rate.

20. Lines 262-278: This paragraph describes how the authors verified that lower T leads to larger R. I appreciate the thoroughness, but is this not an inherent feature of the model? Could it have possibly been otherwise?

The relationship between thresholds and subsequent activity is of course inherent to the definition of TA on the level of single neurons. However, threshold only acts as a modulation, and other effects, e.g. changes in pathway activation, disinhibition, etc., could predominate at the network level. Indeed, looking at Figure 4D/E, the effect is clear, but not so much so as to be blatantly obvious.

21. Lines 279-292: The calculations described in this paragraph, and the reasoning behind them, are very unclear. I would appreciate an explanation of why the measures described there were used and how they were chosen, including why there was weighting by the magnitude of the response difference (was it not “normalizing”? Was the response difference used to multiply or divide the terms?). In line 286, it is not at all clear what the “relative terms” plotted in Fig. 4G-H are. If the quantities described in this section are better explained using formulas, those would be welcome. Please consider coining your terms for these quantities. This would make it simpler to have them explained in more detail in the methods section, and refer to them by name in the results section, with at most a general and short mention of their purpose and nature.

In retrospect, the analysis used here was as impenetrable as its description. We have revised the analysis leading to Figure 4F-H, which the cited paragraph refers to, resulting in a clearer picture of the effect we were trying to highlight without substantially changing the point we were hoping to make.

We have chosen to describe the methods applied in Figure 4 in a subsection (“Contribution of TA to greater activity”) of the “Notation and quantities” part of the Methods, which we refrain from reproducing here due to its length; please see the revised submission.

The revised paragraph now reads as follows:

Yet, as we saw in Figure 4C, the membrane voltages also differed between the two sequences. Since firing is defined as the membrane voltage crossing the firing threshold, differences in either of these two factors could explain differences in activity. Therefore, considering instances of greater activity in dev than msc, we wanted to know to what extent these differences were caused by lower thresholds, rather than higher membrane voltages. To this end, we calculated the relative positive contributions of 𝜃 and 𝑉_𝑚_ to instances (i.e., histogram bins) with greater activity as described in Methods. The resulting histogram is shown in Figure 4F. We see that in most bins, particularly along the response front, the major contribution was provided by 𝑉_𝑚_, as indicated by the blue shading, while greater activity after the main response, as well as during its very early portion, were driven largely by lower 𝜃. A similar picture emerges when controlling for the magnitude of the activity difference and averaging across neurons, as shown in Figure 4G, and is maintained across datasets, as shown in Figure 4H. This indicates that adaptation initiated and dominated the stronger firing at the start of trials, whereas the later response was enlarged primarily as a result of greater presynaptic excitation. In short, lower thresholds kickstarted a self-reinforcing burst of activity.

22. Lines 443-455: This explanation can probably be transferred to the methods section, as it is too technical for the results section.

We moved Eqn 7-8 and the corresponding explanations into the Methods:Notation section, which we accordingly renamed “Notation and quantities”, adjusted the text, and aligned the stimulus variable (“X” in the pre-existing Notation text, “S” around Eqn 7-8) to “S”.

23. Line 449: Where does the value of k = 1.4 mV come from?

This comes from numerical integration; in the described situation (one undepressed excitatory presynaptic spike arriving onto a membrane at rest) causes an EPSP that rises to approximately 1.4 mV above V_rest. This is likely a slight overestimate, since most neurons are at least somewhat depolarized throughout a trial, leading to smaller peak EPSPs.

24. Lines 459-469: The explanation here is hard to follow, somewhat meandering. Please rephrase this passage to make it more clear.

We have dropped the inconsequential remark about response differences between A and B and tightened the remaining descriptions somewhat:

As shown in Figure 8A, we find that the response to A (top) reached the entire network in both sequences, but clearly experienced greater depression in msc than in dev across most of the network. In contrast, the response to B (bottom) in the dev sequence was limited to a small patch near the stimulated area, which consequently experienced stronger depression than in the msc sequence. As a result, the contrast Δ𝑫^B^ was slightly negative in the periphery, but strikingly elevated near the stimulation site, indicating much greater synaptic depression in dev than msc.

25. Lines 474-475: Can the authors explain the situation in the minority of networks, where greater depression in dev did not predict a lower response?

We have answered this in a newly added paragraph:

We believe that the failure of higher D to predict lower responses (in some networks with the full model, and in most networks with STD only) is due in part to the very low strength of depression, with differences between sequences even smaller, and in part to the way D is calculated, which does not exclude silent neurons, nor differentiate between depressed inputs arriving before or after postsynaptic activity. In the latter case, given that most neurons fire at most one spike per trial, the depression is ineffective, but nonetheless counts towards D.

26. Line 477: Why is there no negative dependence in the model with STD only?

The same reasoning applies here.

27. Line 483, and perhaps elsewhere: The phrasing “neurons… showed greater depression…” is somewhat confusing. Perhaps it could be stated in a different way, or given another term rather than simply “depression”, to maintain the distinction that this quantity that is being referred to is a measure for the impact of pre-synaptic depression on a post-synaptic neuron.

We have adjusted the wording for clarity:

We found that, indeed, presynaptic input to neurons responding early to stimulation in B was more depressed in dev than msc (…), while the late portion was subject to slightly less depression (…).

28. Line 502, and elsewhere: “Suppression” in the context of STD is a bit misleading, since there is no active factor suppressing the responses. Please consider alternative terms (e.g. depression, depletion, habituation, adaptation,…). This also applies to TA, in fact. Nevertheless, it is also acceptable to keep “suppression” as it is used now if the authors view it as preferrable to alternative terms.

We agree, and have replaced “suppression” with “fatigue” throughout the manuscript, a term which better captures the nature of the response reduction without biasing readers towards either synaptic *depression* or neuronal *adaptation*, and sidestepping potential confusion with the established concept of habituation in the context of ethology.

29. Lines 505-507: Here, and elsewhere, the authors present the claim that adding STD to a model increases its DDI that is generated by TA. However, I am concerned that this way of things comes about from constructing a network, or using a stimulation paradigm, where STD does not in itself produce DD, contrary to previous modeling work. While the synergy of the two mechanisms that is demonstrated and explained in this present work and mentioned in lines 512-515 is well appreciated, I am wondering if, had the authors started out with a different network or paradigm, they would have reached the opposite conclusion, that it is TA that increases DD when added to a network with STD.

Yes; see discussion along this line of thought on lines 512f (“it is very plausible that TA likewise increases the effect of STD”) and 663ff (“We speculate that increasing the strength of TA, rather than adding STD, would have a similar effect. Thus, prior to saturation, each additional unit of adaptation from any suﬃciently similar source would lead to increasing gains in DD.”)

30. Fig. 9A: Is it correct to infer from the curves that there is no DD at the stimulation site? What is the significance of the network only being able to detect true deviance outside of a stimulus’s representation?

This is correct, adaptation in the neurons under direct stimulation is not strong enough to overcome the input drive. This is perfectly appropriate in the context of neuronal culture, where cells that sit on top of stimulation electrodes likely always respond. In the context of natural networks, the stimulated neurons should perhaps be considered sensory transducers (e.g. cochlear hair cells), with only the rest of the network mapping onto something resembling a processing pathway. This would be consistent also with the unchanging stimulus signal properties, but does of course constitute a weakness in our model.

We have added a brief discussion on the question of stimulation to the new Limitations and future work subsection:

[… T]he manner of stimulus delivery, consisting of an instantaneous, unadaptive excitation that overcomes most or all adaptation in the stimulated neurons, is faithful to the equivalent situation in cell culture, but clearly not representative of e.g. thalamic input to primary sensory cortex.

31. Fig. 9B: What gives rise to the spatial wave seen in B?

The outer two bumps (around 3 and 5 mm) almost certainly derive from the other stimulus locations, which are 2.9 and 4.8 mm away; in particular, we are probably seeing the TA of the neurons under direct stimulation. We think we only see this in TA because of the vast difference between stimulated and unstimulated neurons in this measure.

The nearby bump at 1.5 mm is different: It appears also in unnormalized activity, and indeed even in the recovered response, i.e. in the initial trial. We speculate that this is a very early response driven by the first neurons downstream of the stimulated excitatory neurons; since these project farther than their inhibitory neighbors, the ring between 1 mm and 2 mm from the stimulus site receives a strong excitatory drive without any inhibitory counterbalance in the first few milliseconds. We have added a brief explanation at the end of this subsection, along with a supplementary figure (S8) which shows the unnormalized response:

As a side note, the apparent wave pattern in TA (Figure 9B) likely has two causes. The distal local maxima are only seen in TA and likely reflect high TA levels at the neighboring stimulation sites, located 2.9 and 4.8 mm away. The local maximum around 1.5 mm, by contrast, also appears in unnormalized R (see S8 Fig.) and likely reflects an early response in the band between 1 and 2 mm from the stimulus site, which is reached by excitatory stimulated neurons but not inhibitory due to the connectivity structure.

On reflection, this latter point appears to be a weak link in our story. We had chosen our local/global threshold purely based on the data, and had not realized its relationship to network structure. We do not believe that this is a critical flaw, but we have added a brief discussion on this point:

This band and the chosen distance threshold for the local patch coincide not by conscious choice, yet may raise doubts about the validity of our analysis. However, the gradual decline in R and D beyond the threshold suggests that the principle remains sound, and true DD in our model can indeed by described well by a distance dependence of fatigue.

32. Fig. 9, A-C: What causes the local maxima found just on the left of the divider line in both A, B, and C?

We suspect that this is closely related to the answer above: Up to the 2 mm line, we see the response of the neurons immediately downstream from the stimulated excitatory neurons, which respond more strongly and reliably than any other neurons in the network.

33. Lines 529-541: Do the local and distal subsets correspond to the early- and late-responding neurons from earlier?

The two correspond to some degree, but not entirely. We have added a supplementary figure (S6) that shows the situation in the sample network, along with a note on line 530:

Note that while this overlaps with the "early" subset used above, some early neurons are non-local, and some local neurons respond late or not at all (see S6 Fig.).

Distance to stimulus site A plotted against the response rank (see Figure 6) in the sample network. Vertical and horizontal grey lines represent the cutoffs for the "early" subset (Figure 6) and the "local" subset (Figure 9), respectively. Notice that, while many neurons are both early and local, or both late and global, a substantial number of neurons are early and global, or late and local.

34. Discussion – 1^st^ section and line 554 (and also line 212): I would like to note that “learning” is a term not usually used for the processes happening in neuronal networks (as opposed to artificial neural networks), where the processes underlying learning are referred to by terms like plasticity, adaptation, encoding, etc. Learning would be the process happening on a higher or more abstract level, referring to the way an organism acquires information and is able to use it later on, or adapts to a certain environment or process in a way that changes its behavior. I do not necessarily require the replacement of this word, but please give thought to its use.

We have changed “learning” to “encoding” in the discussion, but decided to retain “learn” on line 212 for rhetorical purposes.

35. Lines 606-608: Were the occasional large responses to std in this model observed in different single neurons on different trials, or were they synchronized responses of the whole population?

We see synchronized large bursts. In the sample network, the response size distribution in std looks like this:

36. Lines 611-612: What is meant by “very minimal tuning and exclusion criteria”?

We made only very few modifications to the initial model design (structure, parameters, stimulation paradigm, etc.; exclusion criteria referring to deselection of insufficiently responsive networks), and focused instead on developing suitable analysis methods. That is, with those methods in hand, we probably could have gone back and polished aspects of the model, either for biological plausibility or to show more convincing results, but, for the purposes of this manuscript, chose not to.

37. Lines 611-612: It is not clear to me why the authors expect that their model has a certain level of robustness, and to what parameters they expect it to be robust. Please elaborate. The parameters chosen here are very specific; I can see how robustness to parameters of the stimulation paradigm (which was not tested, but is easy to test) could easily be explained by the very long tau_theta.

Our intention was to say: We threw TA and a few other features together into a model, and, without putting much effort into searching for it in parameter space, we found true DD. However, of course the choices we made defined the results we got. We have reworked this paragraph substantially to make our point clearer:

Here, we did not explore parameter sensitivity beyond the dependence on stimulus frequency. However, several reasons lead us to believe that our model is relatively robust to parameter choices. Firstly, we did not have to search parameter space to find true DD, but readily found clear evidence for it. Secondly, the spatial organization of our network model is simple and continuous, such that spatial scaling is likely to affect the results quantitatively rather than qualitatively. Thirdly, our structural constraints are mild (e.g., no dependence of connection probability, weight, etc. on postsynaptic neuron type), resulting in a model of low structural complexity and therefore, we surmise, low complexity in its qualitative characteristics. Finally, the dynamic complexity of our network is likewise low, as is evident from the propagation of responses through the network (cf. Figure 4), making it unlikely that our results derive from intricate interactions between model components.

38. Line 613: Is this sentence stating that the present model has looser connectivity requirements than other models, and is thus applicable to a wider range of architectures, or that if the model’s connectivity requirements be made looser, it will be applicable for a wider range of architectures?

The intent was that the present model is comparatively unconstrained, particularly in contrast to models searching for explicit representations of “error” in terms of predictive coding (e.g. Hertäg and Sprekeler, 2020). The revised paragraph quoted above partly addresses this (under “thirdly”). We have further revised the cited sentence as follows:

Given the mild structural constraint in our model noted above, we speculate that our interpretation of the source of true DD is applicable to a wider range of neural architectures, including non-cortical circuitry.

39. Lines 614-616: What structural constraints of their model do the authors consider as significant for the issue of judging what mechanism gives rise to DD? Does their model change its DD properties if the parameters of the lateral inhibition (connectivity of their inhibitory neurons) are changed?
40. Lines 617-618: What feedforward adaptation does the present model include?

Given the response pattern, wherein neurons fire very few spikes, and the response essentially describes a one-time spatial wave, we suspect that lateral inhibition has only little power to affect the outcome. However, this of course depends no small amount on what exactly should be considered “lateral”, “feedforward”, or “feedback” within a continuous, but spatially structured network such as ours. Structurally, the network is highly recurrent, so most, if not all, connections could be considered “lateral”. Dynamically, the response courses through the network like a wildfire, leaving only vestiges of activity in places it has already passed, so by this token, many connections, inhibitory and otherwise, could well be considered feed-forward.

To not go into quite so much detail in the manuscript, we have revised the relevant passage as follows:

On the flipside, the divergence in our model between a heavily recurrent structure on the one hand and a largely one-way response pattern on the other hand makes it difficult to judge whether DD arises as a result of lateral inhibition or adaptation of lateral excitation as in (Yarden and Nelken, 2017; Hertäg and Sprekeler, 2020; Park and Geffen, 2020), as the model structure might indicate, or, conversely, as a result of adaptation in feed-forward connections as in Mill et al. (2011), as the response dynamics might suggest.

41. Lines 686-690: If the chosen values are physiologically plausible, give references and state what parts of the brain the model can plausibly represent. Based on that, the paragraph can be rearranged or rephrased to be less apologetic– using these values is a strength, and the model’s supposed correspondence to physiology can be the outset; then, mention that this choice endows TA with a much stronger effect on STD. On top of that, it can be mentioned that under other conditions the model is likely to generate comparable levels of DD with either mechanism (what conditions? Do they make for a different network, or are these different conditions of stimulation of the same network?).

To be completely frank: Since we did not target a particular system, we put less thought into the choice of time constants than we ought to, in retrospect. The values are perfectly acceptable in general, but may not apply in this particular combination to any known system, hence the defensive tone here. We have rewritten this paragraph as follows:

We believe that the chosen time constants fall within a physiologically plausible range: For synaptic depression, 150 ms, chosen based on thalamocortical inputs to primary sensory areas (David and Shamma, 2013; Pennington and David, 2020), is at the lower end of the plausible range for cortical neurons, with values reported ranging from hundreds of milliseconds to several seconds for intracortical synapses (Tsodyks and Markram, 1997; Markram et al., 1998; Varela et al., 1997; Asari and Zador, 2009; Levy and Reyes, 2011). For threshold adaptation, time constants have been reported in a similar range, from hundred of milliseconds (Henze and Buzsáki, 2001; La Camera et al., 2006; Abolaﬁa et al., 2011; Pozzorini et al., 2015) to several seconds (Schwindt et al., 1989; Fleidervish et al., 1996; Sanchez-Vives et al., 2000a; La Camera et al., 2006). The stark contrast in apparent capabilities between STD and TA in our model, therefore, is likely a result of the choice of time constants. In particular, with the higher time constants for STD reported intracortically, we expect STD to likewise contribute significantly to true DD at longer stimulus intervals.

In revising this passage, we also noticed that the references given for the value of the TA time constant in the Methods section were inappropriate; we have updated these to (Schwindt et al., 1989; Fleidervish et al., 1996; Sanchez-Vives et al., 2000a; La Camera et al., 2006).

42. Lines 698-699: Can the authors give a few examples for such architectures and stimuli?

We have added one:

For example, in primary auditory cortex, where neurons are tuned along a tonotopic gradient, and are located near and connected to similarly tuned neighbors, STD would be better placed than TA to cause a dependence of DD on the pitch separation between presented tones.

43. Lines 708-711: What kind of sensitivity is referred to here, and why does the sensitivity appear to be less than would be expected in this model? On what is the expectation based? And is there a way to test how this model performs with spontaneous activity?

Thank you for pointing this out. By sensitivity, we meant the dependence of DDI or related indices on background activity levels, but we are unsure what our estimate for this in our model could have been. We have removed this remark.

Naturally, yes, we could test the impact of spontaneous activity in our model, but choose to defer this to future work.

44. Line 722: What specific subcortical stations are the authors referring to? Wouldn’t those show DD for stimuli with shorter time intervals between them, a presentation that was not tested with this model?

As far as we know, there is adaptation (in the sense of fatigue) throughout most sensory processing pathways, so we think the principle of local vs. global fatigue applies broadly. We suspect that Reviewer 3 understood “our adaptation-based model” in terms of the particular implementation; we have altered this to read “our conceptual model of local and global fatigue” to avoid such misunderstandings.

The rest of the sentence, previously “is a better description of subcortical processing,” was unclear regardless (better than?), and has been replaced with “may be a valid description of subcortical processing.”

**Minor Comments:**

45. Lines 43-44: What is the difference between statistically surprising sensory inputs and violations of established regularities? Aren’t such violations statistically surprising? Is it meant here that deviance detection is the ability to identify these two things or the ability to distinguish between them? Or are “violations of… “ meant as an alternative description of the previous term (in which case, it would be better to use a comma between them).

This was meant as an alternative description, so we inserted a comma.

46. Line 83: Here, and in other places, the authors use the term “true deviance detection”, while throughout the work they refer to DD, without the adjective “true”. Perhaps it can be clarified that these two terms are used here to refer to the same phenomenon or that “true” is being left out for convenience.

We have clarified the situation with regards to “DD” vs “true DD”; please refer to our response to Reviewer 1 for details.

47. Line 142 and throughout the manuscript: I suggest denoting the units of R as spikes (but leave it to the choice of the authors).

The correct unit description would be “spikes per trial”, as declared in the Notation subsection. Since some figures would get unreasonably cluttered with redundant labels (Fig 7, in particular), we decided to keep this implicit in axis labels.

48. Line 203: Is the response calculated over the whole trial, including the interval between trials, or over a shorter time period?

It is calculated over the entire trial (but most of that is perfect silence).

49. Line 216: This sentence is not clear. Perhaps it was meant that the response “developed earlier and was very slightly larger”?

Fixed.

50. Line 311: in “responded more to…” – more than what? Please clarify.

Fixed:

[…] responded more to non-target stimulation in dev than in msc, […]

51. Line 320: “higher target response” – higher than what? Please clarify.

As above, fixed.

52. Line 329: It would be more clear to replace “in reverse” with a more specific description, or explain it with another clause or in parentheses.

We have decided to break this out into a separate sentence:

Note that the logic here is equivalent to the analysis in Figure 4, where we showed that lower TA in early responders leads to a greater downstream response.

53. Line 344: Does “blue” refer to only some of the neurons in the right plot? If so, please clarify.

It was meant to refer to “everything other than the deepest red”, which is clearly not only blue, even in a two-hued colormap. We have removed “blue” here.

54. Line 411: Please consider rephrasing “differing by 94%” to make this clearer (e.g., X higher than Y by 94%).

We have changed the formulation here and on line 399 to say “X% greater in the full model than in TA only”

55. Lines 414-415: Please be more specific (“increased local suppression” referring to the dev sequence, and “greater target responses” being a comparison to those in msc).

We have added the comparison once, to apply to all four comparatives:

[…] greater local fatigue to B in dev than msc resulting in weaker B responses and less global fatigue, which in turn allows for stronger target responses […]

56. Line 421: Was the effect indeed limited to those trials, or was it just much stronger there?

Note that we are stating a hypothesis here (“We hypothesized that […] the direct effect […] was limited”, current-day English lacks the necessary subjunctive forms to mark this). To the question: We thought (wrongly, as it turned out) that activity in one trial, along with the recovery from depression, would abolish any trace of the preceding trial’s activity.

57. Lines 422-424: As mentioned before, these statements are valid only for the present model and stimulation parameters, as it was already shown that networks with only STD are able to generate DD.

We have qualified this:

This would mean that, in our model, STD is […]

58. Lines 428-429: This sentence is overly complicated (“been able to lead to a build-up”), please rephrase.

Simplified to:

Conversely, the frequent presentation of stimulus B could likely cause enough depression to suppress its own response.

59. Line 431: Consider using “the consecutive” or something similar instead of “later”.

We chose “later” deliberately, since we correlated not just to the immediately following trial; on reflection, “subsequent” works well, too, and is less vague than “later”. Fixed.

60. Line 434: It’s simpler and clearer to refer to decay of adaptation as recovery.

Fixed.

61. Line 538: What is meant by “varied stimulation paradigm”? Please clarify or use a different term.

We have reworded the sentence to be clearer, also with respect to the comparatives:

Incidentally, this greater global fatigue, caused by the greater diversity of stimuli presented in msc than in dev, was also reflected in greater local fatigue […]

62. Line 689: What is meant by “a more even tuning”? Please clarify.

We meant a closer correspondence between the time constants of TA and STD, which seems ill considered in retrospect. This passage has been substantially rewritten; the idea in question is now expressed as follows:

The stark contrast in apparent capabilities between STD and TA in our model, therefore, is likely a result of the choice of time constants. In particular, with the higher time constants for STD reported intracortically, we expect STD to likewise contribute significantly to true DD at longer stimulus intervals.

63. Line 693: The reference to Fig. 10 is not in the regular format.

Nor indeed was it meant to point to Figure 10, which never had a panel C, but rather to Figure 9. Fixed.

**Comments on the Figures and Presentation:**

General:

a) I would advise to increase the size of text in all the figures, including axis labels and legends. Especially, in the sequence names along the y-axis in Fig. 2A.

We have adjusted label sizing throughout all figures.

b) Please consider using a different color for msc rather than blue (perhaps a gold, or green if its brightness is different enough from the red’s). Blue would best be reserved for the standard, as in the literature, blue and red are used for the standard and deviant stimuli, respectively.

We have aligned our color scheme with Nelken lab outputs.

c) In general, the rainbow-like ‘jet’ colormap used throughout is considered less friendly for color-blind readers. Please consider replacing it with an alternative one (e.g. parula, but there are other colormaps that answer the criterion as well).

While we did use matplotlib’s “turbo”, which is less problematic than “jet” in some respects, we acknowledge the problem of colorblind-friendly mapping. We have changed most sequential colormaps from “turbo” to “viridis”, which is perceptually uniform. In Figure 6, we opted for a divergent colormap instead. In order to compensate for the reduced range of distinguishable color levels, we have increased the marker sizes in figures 5 and 6.

d) In many of the figures there are gray lines marking the 0 (or 1, eg. Fig. 2C). Could these lines be made a little less faint?

We have darkened all of these axis helper lines.

e) In general, where the image is not an actual coordinate system (e.g. Fig. 5A-B), please give its title with an upright text, not inclined by 90 degrees like an axis label, as that is easier to read.

We have rotated relevant labels in figures 5 and 8.

Specific:

a) Figure 1A: I believe the axis label of the bottom panel should have a capital V.

Fixed.

b) Figure 1C and 1D: Please use for the sites and stimuli colors or markings that have more contrast between them. The pale colors that were chosen are a bit difficult to distinguish and locate on the already colorful network map for people who have a common form of color blindness. Also, why do the colors of the sites and stimuli appear in different orders in the legends of 1C and 1D? 4 of the colors of 1D seem to resemble the colors of sites in 1C. Can these legends be sorted to have the same order? Or is there no correspondence between the sites of 1C and stimuli of 1D? If there is a correspondence, perhaps the authors could use the same fifth color in both? And if there is no correspondence, please use different colors in 1C and 1D (I know it’s not easy to find several distinct color palettes – here, these include the palette of the colormap used for the single neurons; if that is a problem, then for the stimuli, or for the neurons, one could use different shades of the same color).

We have replaced colors with direct labeling of stimulus locations in panel C to improve readability and to avoid implying any correspondence between panels C and D.

c) Fig. 2: In the last line of the legend, the equation number is missing.

Fixed.

d) Fig. 3B: The asterisks marking the significance are too similar, at least in size, to the outliers. Perhaps they could be made larger or bolder.

We have made the asterisks both larger and bolder.

e) Fig 4: Why are the inhibitory neurons also shown interleaved with the excitatory ones within the colormaps of this figure? Would it make sense to separate the lines in each block to an excitatory and inhibitory block?

We agree that this makes sense, particularly given that the inhibitory population does not have any threshold adaptation. We have adjusted the plot accordingly.

f) Fig. 4D: What is the reason for the bimodal or multimodal distribution of ΔR?

The points clustered around ΔR=0 derive mostly from two groups of neurons: those largely silent in response to stimulation A, even in the dev condition (<0.1 spikes/trial), and those responding very strongly (>1 spike per trial). Excluding these two groups leaves behind a largely unimodal distribution that includes 607 of the 800 excitatory neurons:

See also the new panel D in Figure 2, where we present the full-model data in this way.

g) Fig. 4E: Here and elsewhere, the legend has “[x variable] plotted against [y variable]”; it might be more appropriate to reverse the order to have “[y] plotted against [x]”.

We have changed the captions along these lines in figures 4E, 5D, 7D, 7F, and 8C.

h) Fig. 6: Please consider switching the axes to reflect the fact that ΔR is the dependent variable and ΔΤ the independent variable

Given the later logic, this indeed makes more sense. We have switched the axes in panel B.

i) Fig. 6B: In the legend, “confidence intervals” are mentioned without the percentage (which was mentioned in other occurrences throughout the manuscript).

Fixed, this was of course also 95%.

j) Fig. 6C: What is the reason for calling ΔR a “contrast” rather than simply a difference?

It appears we used “contrast” and “difference” interchangeably, not just here, but elsewhere in the manuscript as well. We have replaced some uses of “contrast” with “difference”, particularly when referring to purely numerical results.

k) Fig. 7, C, E, and G: Please consider switching the axes, as it would make more sense to place the quantities of the TA-only model ad the independent variable and those of the STD+TA model as the dependent variable.

We feel that this would complicate an already complex figure. In particular, notice that the x-axes are shared between panels C and D, and between panels E and F. We feel that this continuity is worth preserving.

l) Fig. 9, legend: In “same data plotted as lines”, consider using “curves” instead of “lines”; for “leaving 120 data points to be summarized…”, consider replacing “leaving” with a different word (e.g “providing”).

We have updated the wording in the caption to Fig 9.

m) Fig. 10: A summarizing figure is definitely appreciated, but this figure is graphically confusing. It’s not clear why the arrows have curved shapes and where they are supposed to point or lead to – both the large arrows and the small ones, which are perhaps too small and thin, and it is not clear why they need to be curved (except for the ones forming a circle in the right-hand panels); having text on top of the arrows is somewhat of a visual load, as are the multiple formats of text that appear here (regular and bold, and different color highlights); and the general layout of text scattered at different positions is also confusing, as it is not clear why these positions are chosen and what they should mean. Some of this confusion is caused by the mixing of a topographical layout with the representation of the abstract, causal relationship between different phenomena. Again, it is excellent to have such a figure closing the manuscript – but the material it is meant to present is unarguably complex. I suggest reaching out to the university’s graphic design services or to an external graphical designer for expert advice or commission of an improved design.

We have replaced the figure with a less busy one that, we hope, illustrates the key concept well.
